# The transcription factor complex CmAP3-CmPI-CmUIF1 modulates carotenoid metabolism by directly regulating the carotenogenic gene *CmCCD4a-2* in chrysanthemum

**DOI:** 10.1093/hr/uhac020

**Published:** 2022-02-19

**Authors:** Chenfei Lu, Jiaping Qu, Chengyan Deng, Fangye Liu, Fan Zhang, He Huang, Silan Dai

**Affiliations:** Beijing Key Laboratory of Ornamental Plants Germplasm Innovation & Molecular Breeding, National Engineering Research Center for Floriculture, Beijing Laboratory of Urban and Rural Ecological Environment, Key Laboratory of Genetics and Breeding in Forest Trees and Ornamental Plants of Education Ministry, School of Landscape Architecture, Beijing Forestry University, Beijing, 100083, China

## Abstract

Carotenoids are one of the most important pigments for the coloring of many plants, fruits, and flowers. Recently, significant progress has been made in carotenoid metabolism. However, our specific understanding of the transcriptional regulation that controls the expression of carotenoid metabolic genes remains extremely limited. Anemone-type chrysanthemums, a special group of chrysanthemum cultivars, contain elongated disc florets in the capitulum that usually differ in color from the ray florets because of their different carotenoid contents. In this study, the carotenoid composition and content of ray and disc florets from the anemone-type chrysanthemum cultivar “Dong Li Fen Gui” were analyzed by high-performance liquid chromatography–tandem mass spectrometry (HPLC–MS/MS), and the key structural gene *CmCCD4a-2*, whose differential expression resulted in different carotenoid contents in these two types of florets, was identified. The promoter sequence of *CmCCD4a-2* was then used as bait to screen a chrysanthemum flower cDNA library, and the transcription factors (TFs) CmAP3 and CmUIF1 were identified. Y2H, BiFC, and Y3H experiments demonstrated that these two TFs were connected by CmPI to form a CmAP3-CmPI-CmUIF1 TF complex. This TF complex regulated carotenoid metabolism by directly activating the expression of *CmCCD4a-2*. A large number of target genes regulated directly by the CmAP3-CmPI-CmUIF1 TF complex, including carotenoid biosynthetic genes, flavonoid biosynthetic genes, and flower development-related genes, were identified by DNA-affinity purification sequencing (DAP-seq). This result indicated that the CmAP3-CmPI-CmUIF1 TF complex may participate in multiple processes. These findings expand our knowledge of the transcriptional regulation of carotenoid metabolism in plants and will be helpful for manipulating carotenoid accumulation in chrysanthemum.

## Introduction

Carotenoids, a group of important secondary metabolites that make flowers and fruits appear in diverse colors, are biosynthesized and stored in all photosynthetic organisms, including algae and plants [1]. In the green tissues of plants, carotenoids are involved in the capture of light energy, transferring it to chlorophyll for photosynthesis. They also participate in photoprotection [2] and serve as precursors for phytohormone molecules [3]. Besides their physiological functions in plants, carotenoids are also essential for human nutritional balance and health [4, 5]. It is extremely important to clarify the regulation of carotenoid metabolism for the cultivation of horticultural crops with high nutritional value.

Carotenoid metabolic genes have been identified extensively in many plants through biochemical analysis and molecular biology [6]. Major enzymes in the carotenoid metabolic pathway include deoxyxylulose 5-phosphate synthase (DXS), deoxyxylulose 5-phosphate reductoisomerase (DXR), phytoene synthase (PSY), phytoene desaturase (PDS), ζ-carotene desaturase (ZDS), and carotene isomerase (CRTISO), which mainly participate in the synthesis of linear carotenes such as phytoene, zeta-carotene, and lycopene. Lycopene represents the branch point of the carotenoid biosynthetic pathway and can be cyclized by lycopene β-cyclase (LCYB) and lycopene ε-cyclase (LCYE) to produce α- or β-carotene, which can then be hydroxylated and epoxidated to produce various xanthophylls. Both carotenoid biosynthesis and degradation determine the steady-state level of carotenoids in plants [7]. Thus, the catalytic function of carotenoid cleavage dioxygenases (CCDs), which cleave carotenoids to produce apocarotenoids and their derivatives, such as phytohormones (ABA and SLs) and signaling molecules, is critical for manipulating carotenoid accumulation. Previous research has shown that members of the CCD1 and CCD4 subfamilies can affect the carotenoid content in organs of various plant species, including the fruits of peach and the flowers of chrysanthemum and osmanthus [8–10]. Many studies have reported that the expression levels of these carotenoid metabolic genes are closely associated with carotenoid accumulation in plants [7]. However, the specific transcriptional regulation that controls the expression of these structural genes remains poorly understood.

Transcription factors (TFs) are key regulatory factors in plant growth and development that bind directly to cis-acting elements of downstream target genes to regulate their expression. Currently, many TFs that directly modulate carotenoid metabolic processes have been characterized [6]. For example, PHYTOCHROME-INTERACTING FACTOR 1 (PIF1) repressed the transcription of *AtPSY*, a key structural gene encoding a major flux-controlling enzyme in carotenoid metabolism, by binding directly to the G-box element in *Arabidopsis* [11]. The transcription factor RAP2.2 from the AP2/ERF family can bind specifically to the promoters of *AtPSY* and *AtPDS* to regulate their expression [12]. Other TFs, including CpNAC1 from papaya [13], MtWP1 from *Medicago* [14], and AdMYB7 from kiwifruit [15] are also involved in the regulation of carotenoid metabolism.

MADS-box transcription factors are of ancient origin and are found in animals, fungi, and plants. They participate in various developmental processes in plants, including vegetative organ development, flowering, floral organ identity, fruit ripening, and metabolism [16]. Recently, several MADS-box ripening regulators, including RIPENING INHIBITOR (RIN), tomato AGAMOUS-LIKE1 (TAGL1), FRUITFULL1 (FUL1), and FUL2, have been identified in tomato fruits; they can form dimeric and tetrameric complexes to affect carotenoid metabolism [17, 18]. In citrus, CsMADS5 was characterized as a positive regulator that interacted with CsMADS6 to synergistically promote carotenoid accumulation [19]. AP3 and PI belong to the class B DEFICIENS/APETALA3 and GLOBOSA/PISTILLATA subfamilies of MADS TFs, respectively, and many studies have reported the roles of AP3 and PI homologs in floral organ identity [20, 21]. The GARP transcription factor family, which is composed of *Golden2-like*, *ARR-B,* and *Psr1*, is mainly involved in hormonal signaling, nutrient response and sensing processes, chloroplast biogenesis, and plant development [22]. Recently, the GARP TF UIF1 (ULT1 Interacting Factor 1), which can bind directly to the promoters of *WUS* and *AG* to exert regulatory functions on petal number, was identified in *Arabidopsis* [23]. However, there are no reports on the interaction between AP3, PI, and UIF1, and the exact roles of these TFs in carotenoid metabolism are still unclear.

*Chrysanthemum × morifolium*, one of the top ten well-known traditional Chinese flowers, is a leading flower with applied value worldwide [24]. As the most representative ornamental flower in Asteraceae, chrysanthemum has a typical capitulum composed of ray florets and disc florets. Anemone-type chrysanthemums, a special group of chrysanthemum cultivars, contain elongated disc florets in the capitulum, which have the same ability as the ray florets to accumulate pigments [25]. The elongated disc florets of almost all anemone-type chrysanthemum cultivars appear yellow because they accumulate large amounts of carotenoids, whereas the ray florets may appear white, pink, yellow, orange, or red because they accumulate different levels of carotenoids and anthocyanins simultaneously [26]. Thus, these two types of florets in anemone-type chrysanthemums are excellent materials for studying the regulatory mechanism of carotenoid metabolism. In this study, we identified the key structural gene *CmCCD4a-2*, whose differential expression resulted in the different carotenoid contents of ray and disc florets of the anemone-type chrysanthemum cultivar “Dong Li Fen Gui”. We then characterized the TF complex CmAP3-CmPI-CmUIF1 and found that it regulated carotenoid metabolism by directly activating the expression of *CmCCD4a-2*. Other downstream target genes regulated by this TF complex were also identified by DNA-affinity purification sequencing (DAP-seq). These findings expand our understanding of the transcriptional regulation of carotenoid metabolism in plants and will be helpful for controlling carotenoid accumulation in chrysanthemum.

## Materials and methods

### Plant materials

**Figure 1:**
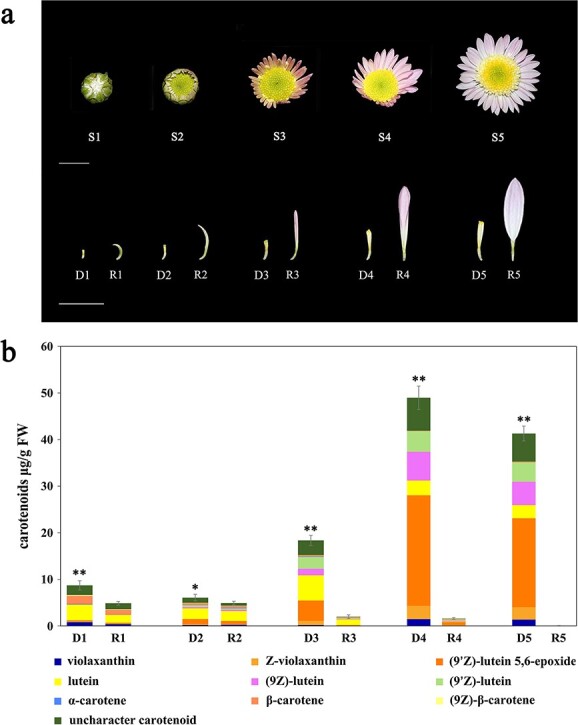
Characteristics of capitulum developmental stages of “Dong Li Fen Gui” and analysis of carotenoid components and contents. **a.** The development of the capitulum of “Dong Li Fen Gui” chrysanthemum was divided into five stages (S1–S5). R1–R5 and D1–D5 represent ray florets and disc florets of “Dong Li Fen Gui” at different developmental stages. Bar, 1 cm. **b.** Carotenoid components and contents of ray florets (R1–R5) and disc florets (D1–D5) of “Dong Li Fen Gui” at different developmental stages. The data are presented as the mean ± SD from at least three biological replicates (^*^P < 0.05; ^**^P < 0.01; Student’s *t*-test).

The chrysanthemum cultivar “Dong Li Fen Gui”, an anemone-type chrysanthemum, contains elongated disc florets in the capitulum whose colors differ from those of the ray florets because of their distinct carotenoid contents (Fig. 1a). This cultivar was the main material used to screen key carotenoid metabolic genes and study their transcriptional regulatory mechanism. Other chrysanthemum cultivars from different color groups (white group: “404 × C34–79”, “404 × C34–85”; pink group: “317”, “B200”; yellow group: “404”, “404 × C60–10”; red group: “A49”, “D91”) (Fig. S4a) were selected to verify the expression patterns of key structural genes in ray and disc florets. All plant materials were grown in the chrysanthemum germplasm nursery of the Beijing Forestry University. The capitulum development of “Dong Li Fen Gui” was divided into five stages (S1–S5) based on Lu et al. 2019 [27]. The ray florets (R1–R5) and disc florets (D1–D5) of “Dong Li Fen Gui” at different developmental stages (Fig. 1a) were collected for carotenoid component and content analysis, comparative transcriptomics, and gene expression profiling.

### Extraction and analysis of carotenoids by HPLC–MS/MS

Carotenoid components and contents of the ray florets and disc florets of “Dong Li Fen Gui” at different developmental stages were analyzed by HPLC**–**MS/MS. First, 0.25 g of tissue was weighed and added to 4 ml pigment extract. Supernatants were transferred into new 10-ml centrifuge tubes and dried under nitrogen (<30°C). The dried carotenoid extracts were dissolved in 2 ml MTBE, then saponified in 2 ml of 10% KOH-methanol solution for 10 h away from light. After saponification, 4 ml NaCl solution and 2 ml MTBE were added to the sample, and the supernatant (MTBE-carotenoid solution) was concentrated to dryness under nitrogen once again. The dried carotenoid extracts were dissolved thoroughly in 0.6–1 ml MTBE-methanol solution (1:1, v/v), and the supernatants were filtered through a 0.22-μm membrane filter to prepare the sample for HPLC detection. The HPLC elution program was published previously by Huang et al. 2019 [28]. LC**–**MS/MS was performed on the same HPLC as above. Carotenoid components were identified using standards (β-carotene, violaxanthin, and lutein) (Sigma), the specific absorption wavelength, and the retention times and MS data of carotenoids reported in the published literature [27, 29].

### RNA sequencing, functional annotation, and data processing

RNA sequencing (RNA-seq) has been widely used to screen out key structural genes and TFs in plants. In this study, the ray florets (R3) and disc florets (D3) at the S3 stage were sampled for RNA-seq. In brief, total RNA was extracted from R3 and D3, and cDNA libraries were synthesized. The obtained libraries were sequenced on the Illumina HiSeq 2500 platform (Illumina, USA). After adaptor sequences and low-quality sequences were removed, the clean reads were assembled into unigenes using SOAPdenovo software with the parameters -K29, −M2, −L50. The transcript abundances of all unigenes were estimated via the FPKM method using RSEM [30]. To obtain the key carotenoid metabolic genes, differentially expressed gene (DEG) analysis was performed with the DESeq package using the parameters false discovery rate (FDR) < 0.01 and fold change (FC) ≥ 2. We then used Blast2GO with the cut-off of E-value <1 × 10^−5^ to classify DEGs into functional categories and assigned a candidate ko number by searching the KEGG pathway database [31].

### Gene expression analysis by semi-quantitative RT-PCR and quantitative real-time PCR

Semi-quantitative reverse transcription polymerase chain reaction (RT-PCR) was performed to analyze the expression profiles of all carotenoid metabolic genes in ray and disc florets of chrysanthemum. First, total RNA was extracted from ray (R1–R5) and disc florets (D1–D5) of “Dong Li Fen Gui”. Subsequently, first-strand cDNA was synthesized with a transcription kit for semi-quantitative RT-PCR. The procedure for semi-quantitative RT-PCR followed the method of a previous study [27]. The expression levels of carotenoid metabolic genes were detected, including *CmDXS* (EVM0005576), *CmDXR* (EVM0031358)*, CmIPI* (EVM0051920), *CmGGPS* (EVM0014663), *CmPSY1* (EVM0027963), *CmPSY2* (EVM0033788), *CmPDS* (EVM0005023), *CmZDS* (EVM0015447), *CmLCYB* (EVM0028157), *CmLCYE* (EVM0039265), *CmCHYB* (EVM0048287), *CmCHYE* (EVM0040022), *CmVDE* (EVM0007951), *CmZEP* (EVM0021607), *CmCCD1* (EVM0003018), and *CmCCD4a-2* (EVM0019666). We quantified the intensity of the electrophoretic bands obtained from semi-quantitative RT-PCR using the Tanon GIS system and used these data to construct a heatmap with BMKCloud to visually display the expression patterns of the carotenoid metabolic genes.

Quantitative real-time PCR (qRT-PCR) was performed to analyze the expression patterns of key carotenoid metabolic genes. According to the instructions of the SYBR Premix Ex Taq kit (Japan, Takara), qRT-PCR analysis was performed on a CFX96 real-time system (Bio-Rad Laboratories, USA). Relative expression levels were calculated using the 2^−ΔΔCt^ method [32]. As *β-actin* is stably and constitutively expressed in most tissues and cells, it has been widely used as a reference gene in rice (*Oryza sativa*) [33], maize (*Zea mays*) [34], and wheat (*Triticum aestivum*) [35]*.* In this study*,* we regarded *β-actin* as the preferred reference gene for semi-quantitative RT-PCR and qRT-PCR analysis*.* To further verify the accuracy of the expression patterns, we also used *SAND* (SAND family protein) and *F-box* (F-box protein), which have been reported as stably expressed genes in chrysanthemum [36, 37], as new reference genes for qRT-PCR normalization. The specific primer sequences used for semi-quantitative RT-PCR and qRT-PCR are listed in Table S1.

### Yeast one-hybrid screening

The promoter of the *CmCCD4a-2* gene was inserted into the pAbAi vector to form the bait construct pAbAi-*CmCCD4a-2* promoter. Subsequently, the bait construct was integrated into the Y1HGold yeast strain to create a bait strain including the promoter sequence of *CmCCD4a-2*. This bait yeast strain was plated on SD/−Ura medium and SD/−Ura + 100/150/200/300 ng/ml Aureobasidin A (AbA) media to determine the minimum inhibitory concentration of AbA. SMART cDNA synthesis technology was then used to construct a cDNA library from the two types of chrysanthemum florets. This cDNA library was transformed into the bait yeast strain and inoculated on SD/−Leu + 200 ng/ml AbA medium for selection.

For the Y1H assay, the coding sequences (CDSs) of *CmUIF1*, *CmPI*, and *CmAP3* were inserted into the pGADT7 plasmid to produce the recombinants AD-*CmAP3*, AD-*CmPI*, and AD-*CmUIF1*, respectively. The above recombinants and pGADT7 were transformed into bait yeast strains integrating pAbAi-*CmCCD4a-2* promoter or the empty pAbAi vector (as a negative control). The transformed yeast strains were dotted onto SD/−Leu medium and SD/−Leu + 200 ng/ml AbA medium. Interactions between TFs and the bait sequence were observed after 2 days of incubation at 30°C.

### Gene isolation and phylogenetic analysis

The CDSs of *CmAP3*, *CmPI*, and *CmUIF1* were amplified, and the sequences were aligned by the MUSCLE algorithm in MEGA X with amino acid sequences from *Arabidopsis*, gerbera, and other plants. Based on the maximum likelihood (ML) estimation method, phylogenetic trees of *CmAP3, CmPI*, and *CmUIF1* were constructed. Tree nodes were evaluated with 2000 bootstrap replicates. Evolutionary distances were computed using the p-distance method. After eliminating gaps and missing data, phylogenetic trees of *CmAP3*, *CmPI*, and *CmUIF1* were constructed.

### Subcellular localization

The CDSs of *CmUIF1*, *CmPI*, and *CmAP3* were amplified and recombined into pBI121-eGFP to produce *CmUIF1-GFP*, *CmPI-GFP*, and *CmAP3-GFP* constructs. *Agrobacterium tumefaciens* strain GV3101 transformed with the above recombinants or pBI121, which served as a negative control, were infiltrated into the leaves of *Nicotiana benthamiana*. A confocal laser scanning microscope (Leica TCS SP8, Wetzlar, Germany) was used to record the expression of GFP at 48 hours after infiltration.

### Yeast two-hybrid assay

The CDSs of *CmAP3*, *CmPI*, and *CmUIF1* were recombined into either pGADT7 or pGBKT7 to form the prey recombinants AD-*CmAP3*/AD-*CmPI*/AD-*CmUIF1* or the bait recombinants BD-*CmAP3*/BD-*CmPI*/BD-*CmUIF1*. The various prey and bait recombinants were co-transformed into the Y2H strain, and the transformed yeasts were dotted onto SD/−Trp/−Leu medium, SD/−Trp/−Leu/–His/−Ade+3AT medium, and SD/−Trp/−Leu/−His/−Ade+3AT + X-$ \alpha $-Gal medium. Two proteins were considered to interact with each other if the transformed yeast cells grew well on all media and turned blue on selective medium with X-$ \alpha $-Gal.

### Bimolecular fluorescence complementation assay

BiFC assays were performed to confirm the interactions between CmAP3, CmUIF1, and CmPI TFs. The CDSs of *CmPI* and *CmUIF1* were recombined into pSPYCE to generate YCE-CmPI and YCE-CmUIF1 constructs. The CDSs of *CmAP3* and *CmUIF1* were inserted into pSPYNE173. Then GV3101 containing the above recombinants or the empty vectors pSPYCE and pSPYNE173 were co-infiltrated into the leaves of *N. benthamiana*. The fluorescence signals were examined with a laser scanning confocal microscope at 48 hours after infiltration (Leica TCS SP8, Wetzlar, Germany).

### Yeast three-hybrid assay

As CmPI can form heterodimers with CmAP3 and CmUIF1, a Y3H assay was performed to determine whether CmAP3, CmPI, and CmUIF1 would form a protein complex essential for transcriptional regulation. First, the CDS of *CmPI* was amplified and inserted into the MCS II site of the pBridge plasmid expressed only in the absence of methionine to produce the pBridge-*CmPI* construct. The CDSs of *CmAP3* and *CmUIF1* with *Eco*RI and *Bam*HI restriction sites were cloned into the MCS I site of the pBridge-*CmPI* construct to obtain the pBridge-*CmPI*-*CmAP3* and pBridge-*CmPI*-*CmUIF1* constructs, respectively. The construct pBridge-*CmPI*-*CmAP3*/pBridge-*CmPI*-*CmUIF1* was co-transformed with pGADT7 or AD-*CmUIF1*/AD-*CmAP3* into the yeast strain Y2H and incubated on SD/−Leu/−Trp medium. Finally, the transformed yeast strains were dotted onto SD/−Trp/−Leu + 200 ng/ml AbA medium to assess the CmAP3 and CmUIF1 interaction without the presence of CmPI and onto SD/−Trp/−Leu/−Met+200 ng/ml AbA medium to assess the CmAP3 and CmUIF1 interaction in the presence of CmPI.

### Dual luciferase reporter assay

A dual luciferase reporter (DLR) assay was performed to detect transcriptional activation of target promoters by the AP3-PI-UIF1 TF complex. The full-length sequences of *CmAP3*, *CmPI*, and *CmUIF1* were recombined into the overexpression plasmid pGreenII0029 62-SK, and the resulting constructs were named SK-*CmAP3,* SK-*CmPI*, and SK-*CmUIF1*. The promoter sequence of *CmCCD4a-2* was amplified from genomic DNA and inserted into pGreenII 0800-LUC. GV3101 that contained SK-*CmAP3* or/and SK-*CmPI* or/and SK-*CmUIF1* were mixed with the LUC-*CmCCD4a-2* promoter (10:1; v:v), and the mixture was infiltrated into the leaves of *N. benthamiana*. Luciferase activity was assayed with the Dual-luciferase Reporter Assay System (Promega) on a PerkinElmer EnVision plate reader.

### DNA-affinity purification sequencing

DAP-seq was performed according to the procedure published by O’Malley et al. [38] and Bartlett et al. [39]. First, genomic DNA was extracted from the leaves and flowers of *Chrysanthemum nankingense,* one of the key progenitors of domesticated chrysanthemum [40] using the DNeasy Plant Mini Kit (Qiagen, Germany). The gDNA was broken into fragments with an average of 200 bp, and a short DNA sequencing adaptor was attached. The CDSs of *CmAP3*, *CmPI*, and *CmUIF1* were cloned into the HaloTag expression vector to generate recombinants. *Escherichia coli* strains containing these recombinants were cultivated, and the fusion proteins were purified with Magne HaloTag Beads (Promega, USA). A mixture of Halo-CmAP3/CmPI/CmUIF1 and the Magne HaloTag Beads was incubated with 200 ng of fragmented gDNA on a rotator for 1 h at room temperature. After incubation, gDNA fragments enriched from the beads were amplified by PCR and sequenced on the Illumina NovaSeq 6000 platform. DAP-seq peaks were identified using MACS2 with default parameters. BEDTools was used to detect the distribution of DAP-seq peaks at the whole-genome level. Motif discovery was performed using the MEME-ChIP suite 5.0.5 [41].

### Statistical analysis

All data are presented as the mean ± SD (error bars indicate the standard deviations of the means) from at least three biological replicates. Statistical analysis was performed by Student’s *t*-test, and significant differences between means were defined as ^**^P < 0.01 and ^*^P < 0.05.

## Results

### Carotenoid composition and content of ray and disc florets of “Dong Li Fen Gui”

Carotenoids are a group of important pigments that affect the flower color of chrysanthemum [26]. HPLC–MS/MS was used to clarify whether the different flower colors of ray and disc florets of “Dong Li Fen Gui” were caused by the accumulation of different levels of carotenoids. We found that both ray and disc florets were able to accumulate carotenoids with similar content and composition, including mainly violaxanthin, lutein, and β-carotene, at the early developmental stage of the capitulum (S1) (Fig. 1b). As the capitulum developed, the total carotenoids of the ray florets gradually decreased, and only trace amounts were present at the S5 stage (0.025 μg/g). By contrast, the total content of carotenoids increased significantly in disc florets, reaching a maximum of 48.974 μg/g at the S4 stage and 41.295 μg/g at the S5 stage, 4.960- and 4.182-fold higher than that at the S1 stage. Lutein and its isomers such as (9*Z*)-lutein, (9′*Z*)-lutein, and (9′*Z*)-lutein-5,6-epoxide were the main carotenoid components in the disc florets at the S4 and S5 stages, accounting for 76.41% and 75.17% of total carotenoids, respectively (Fig. 1b).

**Figure 2:**
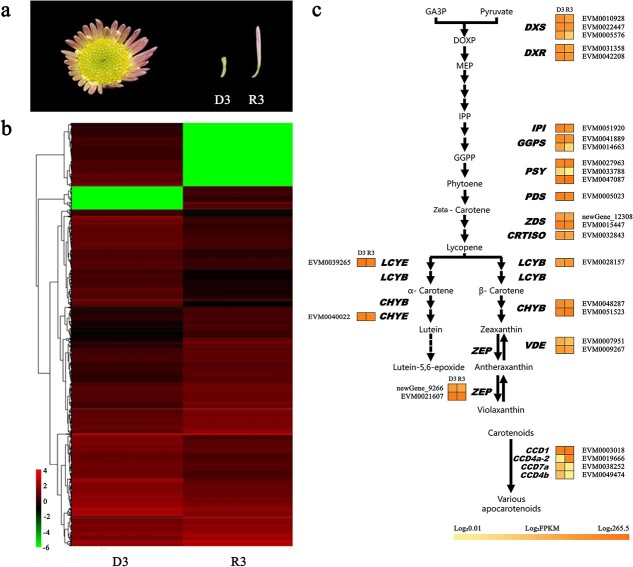
Key carotenoid metabolic genes identified based on comparative transcriptomics analysis. **a**. The ray and disc florets of “Dong Li Fen Gui” at the S3 stage were used for RNA sequencing. **b**. Heatmap analysis of all obtained genes based on the transcriptome data. C. Transcripts of all carotenoid metabolic genes in ray and disc florets of “Dong Li Fen Gui” at the S3 stage based on the transcriptome data.

### Screening for candidate carotenoid metabolic genes by comparative transcriptomics and gene expression analysis

To screen the key carotenoid metabolic genes whose differential expression resulted in the different carotenoid contents of ray and disc florets, RNA samples from these two types of floret in “Dong Li Fen Gui” at the S3 stage were analyzed by RNA-seq (Fig. 2a-b). A total of 54 603 unigenes were assembled and annotated against public databases: Nr (38 527/54 603; 70.55%), COG (17 315/54 603; 31.71%), Pfam (41 697/54 603; 76.36%), KOG (29 690/54 603; 54.37%), Swiss-Prot (38 285/54 603; 70.11%), KEGG (19 807/54 603; 36.27%), and GO (29 534/54 603; 54.09%). The transcriptome data of the two floret types were compared (R3 vs. D3), and 4329 DEGs were identified (Fig. S1a). Gene ontology (GO) annotation was performed to clarify the functions of the DEGs. The 4329 DEGs were classified into 20, 16, and 15 categories and assigned to biological processes, cellular components, and molecular functions (Fig. S1b-c). KEGG enrichment analysis was carried out to further understand the biological functions of these DEGs through pathway classification and enrichment. The DEGs showed the greatest enrichment in pathways such as starch and sucrose metabolism (ko00500), carbon metabolism (ko01200), and biosynthesis of amino acids (ko01230). Some DEGs, such as *CmDXS* (EVM0005576), *CmVDE* (EVM0007951), and *CmCCD4a-2* (EVM0019666), were also enriched in carotenoid biosynthesis (ko00906) (Fig. S1d-e).

**Figure 3:**
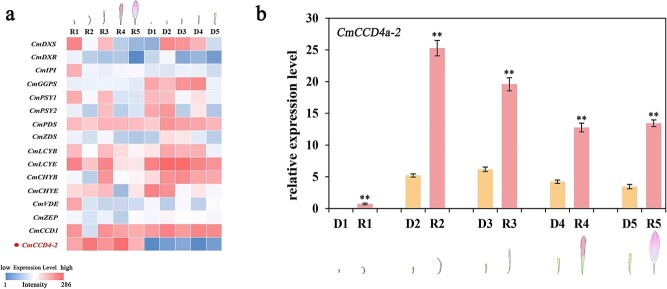
Gene expression analysis of *CmCCD4a-2* in the ray and disc florets of “Dong Li Fen Gui” at different developmental stages. **a.** Identification of key carotenoid metabolic genes that caused differences in the carotenoid contents of ray and disc florets of “Dong Li Fen Gui” by heatmap analysis using semi-quantitative RT-PCR data. **b**. The expression profile of *CmCCD4a-2* was analyzed by qRT-PCR. All data are presented as the mean ± SD from at least three biological replicates (^*^P < 0.05; ^**^P < 0.01; Student’s *t*-test).

To validate the RNA-seq data and identify the key structural genes, all carotenoid metabolic genes were obtained (Fig. 2c), and semi-quantitative RT-PCR was performed to measure their expression patterns during capitulum development. Only *CmCCD4a-2,* which encodes a carotenoid cleavage dioxygenase in chrysanthemum, was differentially expressed between ray florets and disc florets at all developmental stages. This key structural gene was highly expressed in ray florets of “Dong Li Fen Gui”, whereas it showed almost no expression in the disc florets, showing a negative correlation with carotenoid accumulation (Fig. 3a; Fig. S2); qRT-PCR verified this result (Fig. 3b; Fig. S3). Sequence alignment and phylogenetic analysis showed that CmCCD4a-2 had high homology with CmCCD4a*,* which causes the ray florets of chrysanthemum to appear white by cleaving carotenoids into colorless compounds [9] (Fig. S4). Thus, we regarded *CmCCD4a-2* as the key carotenoid metabolic gene responsible for the difference in carotenoid content between ray and disc florets of “Dong Li Fen Gui”. Next, chrysanthemum cultivars from different color groups (Fig. S5a) were used to determine whether *CmCCD4a-2* had the same expression pattern in the ray and disc florets of other chrysanthemum cultivars. By analyzing carotenoid content and gene expression, we found that large amounts of carotenoids accumulated in the disc florets of white- and pink-colored chrysanthemum cultivars, whereas only low transcript levels of *CmCCD4a-2* were detected. By contrast, *CmCCD4a-2* was highly expressed in ray florets, which contained only trace amounts of carotenoids (Fig. S5b-c; Fig. S3). These results indicated that *CmCCD4a-2,* whose differential expression caused differences in carotenoid accumulation between ray and disc florets, was the most important carotenoid metabolic gene in chrysanthemum.

### Yeast one-hybrid screening and identification of candidate TFs

**Figure 4:**
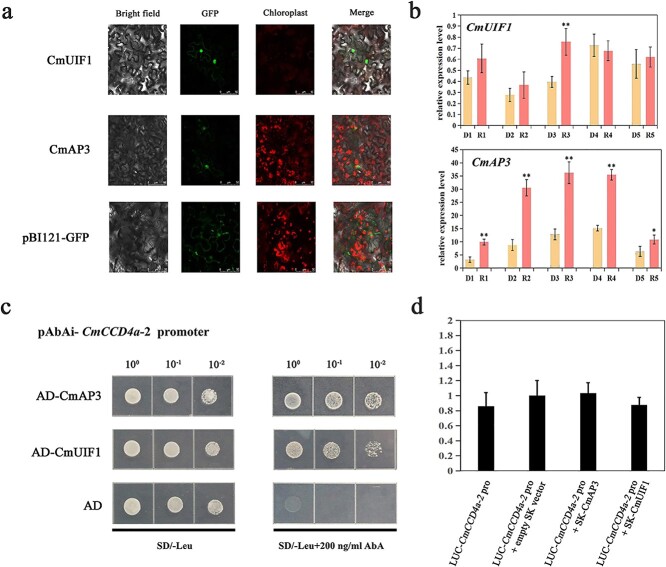
Identification of the candidate upstream transcription factors CmAP3 and CmUIF1. **a.** Subcellular localization of CmAP3 and CmUIF1 in tobacco leaves. The green GFP fluorescence signal was imaged by a laser scanning confocal microscope 48 hours after injection. **b.** The expression profiles of *CmAP3* and *CmUIF1* were analyzed by qRT-PCR. **c.** Y1H showed the binding of CmAP3 and CmUIF1 to the *CmCCD4a-2* promoter. **d.** Effect of CmAP3 and CmUIF1 on the activity of the *CmCCD4a-2* promoter by dual-luciferase assay. All data are presented as the mean ± SD from at least three biological replicates (^*^P < 0.05; ^**^P < 0.01; Student’s *t*-test).

Y1H screening was performed to identify TFs that directly regulated the expression of *CmCCD4a-2*. First, the 1352-bp sequence upstream of the “ATG” of *CmCCD4a-2* was isolated from “Dong Li Fen Gui” by the genome walking method. As the TATA-box is a core promoter element and is close to the transcription initiation site, the sequence from the 5′ end (“ACCCTTCAA”) to the TATA-box was defined as the promoter of *CmCCD4a-2* (Fig. S6a). Possible cis-acting elements, including the ABA-responsive ABRE motif, MYB-binding motif, MYC motif, GARP binding motif, and MADS-box binding motif, were predicted using the PlantCARE and PlantPAN 3.0 databases (Fig. S6a-b; Table S2). This promoter sequence was integrated into the Y1HGold yeast strain to create a bait yeast strain. We plated the bait yeast strain onto SD/–Ura medium with 100/150/200/300 ng/ml AbA and found that 200 ng/ml AbA was the minimum inhibitory concentration for the pAbAi-*CmCCD4a-2* promoter bait strain (Fig. S7). Subsequently, a cDNA library was transformed into this bait yeast strain, and it was inoculated onto SD/−Leu + 200 ng/ml AbA medium for selection. Thirteen positive clones were obtained successfully through Y1H screening, and two proteins (CmAP3 and CmUIF1) were identified and regarded as candidate TFs that might bind directly to the promoter of *CmCCD4a-2* (Table S3). Phylogenetic analysis revealed that CmAP3 belonged to the class B DEFICIENS/APETALA3 subfamily of MADS TFs and was closely related to the *C. lavandulifolium* AP3 protein (Fig. S8a). Through multiple sequence alignment, we found that CmAP3 contained highly conserved domains (MADS domain and K-box region) that are typical of the MADS TFs (Fig. S8c). CmUIF1 from the Golden 2-like subfamily of GARP TFs was highly homologous to AtUIF1 (Fig. S8b, S8d), which modulates flower development by binding directly to the *WUS* and *AG* promoters in *Arabidopsis* [23]. Next, the subcellular localizations of GFP fusions with CmAP3 and CmUIF1 were examined. All GFP fluorescence was detected in the nucleus (Fig. 4a), indicating that the CmAP3 and CmUIF1 proteins, as typical transcription factors, were localized in the nucleus. Gene expression analysis by qRT-PCR showed that *CmAP3* was expressed at higher levels in ray florets than in disc florets of “Dong Li Fen Gui”, whereas the expression level of *CmUIF1* did not differ significantly between these two types of floret (Fig. 4b; Fig. S3).

**Figure 5:**
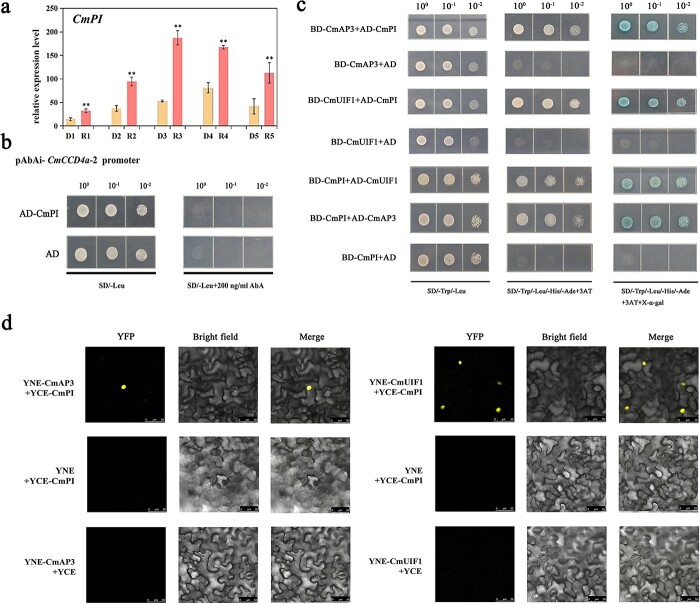
Identification of the intermediate “bridge” protein CmPI. **a.** The expression profile of *CmPI* was analyzed by qRT-PCR. The data are presented as the mean ± SD from at least three biological replicates (^*^P < 0.05; ^**^P < 0.01; Student’s *t*-test). **b.** Y1H assay showing that CmPI was not able to interact with the *CmCCD4a-2* promoter. **c.** Protein interaction analysis between CmPI and CmAP3/CmUIF1 by Y2H assay. **d.** A BiFC assay confirmed the interaction between CmPI and CmAP3/CmUIF1 in *Nicotiana benthamiana* leaves. The YFP fluorescence signal was imaged by a laser scanning confocal microscope 48 hours after injection.

Y1H assays were performed to detect the interaction between CmAP3/CmUIF and the *CmCCD4a-2* promoter. We found that bait yeast cells containing either AD or the recombinant vector AD-CmAP3/AD-CmUIF1 grew well on synthetic dropout medium without leucine (SD/−Leu). Only the bait yeast cells containing the recombinant vector AD-CmAP3 or AD-CmUIF1 survived on selective medium supplemented with 200 ng/mL AbA (SD/−Leu + 200 ng/ml AbA) (Fig. 4c). AD-CmAP3 and AD-CmUIF1 were also transformed into the Y1HGold yeast strain integrating the empty pAbAi vector (as a negative control), and these transformed yeast cells did not grow well on SD/−Leu + 200 ng/ml AbA medium (Fig. S9), indicating that the interaction between CmAP3 and CmUIF1 with the *CmCCD4a-2* promoter was specific and functional. To identify the binding sites of CmAP3 and CmUIF1, all the MADS-box and GARP binding motifs predicted by PlantCARE and PlantPAN 3.0 (Fig. S6a-b; Table S2) were compared with the binding sites of AtAP3 (*Arabidopsis*), AqAP3–3 (*Aquilegia coerulea*), and AtUIF1 identified from ChIP-seq data. These reported CHIP-seq data indicated that CArG boxes, including C(A/T)_8_G and CC(A/T)_6_GG, could recruit the AP3 TF in *Arabidopsis* [42] and columbine [43], whereas UIF1 preferred to bind the motif (AGA(A/T)TC) in *Arabidopsis* [23]. Thus, we speculated that CmAP3 and CmUIF1 TFs might interact with the *CmCCD4a-2* promoter by binding to the CArG box (“CTTAAAAAAG”; “CTTAAATAAG”) and the UIF1-binding motif (“AGAATC”; “AGATTC”), respectively. However, dual luciferase assays showed that single infiltration of CmAP3 or CmUIF1 did not induce any *CmCCD4a-2* promoter activity (Fig. 4d). Transcription factors often perform regulatory functions by forming protein complexes [7, 19], and a Y2H assay was carried out to test the protein–protein interaction (PPI) between CmAP3 and CmUIF1. Yeast cells containing both CmAP3 and CmUIF1 recombinant vectors were not able to grow on selective medium supplemented with 3AT and X-α-gal, as shown in Fig. S10a, indicating that CmAP3 did not interact directly with CmUIF1. A BiFC assay verified this result (Fig. S10b).

### CmPI, an intermediate “bridge” protein connecting CmAP3 and CmUIF1

As one of the most important TFs determining floral organ identity, PI tends to interact with AP3 to form heterodimers that perform regulatory roles [21]. PI, AP3, and UIF1 can all regulate petal development in plants [20, 23]. We speculated that PI might act as an intermediate protein that connects CmAP3 and CmUIF1. To verify this hypothesis, the full-length sequence of *CmPI* was cloned, and phylogenetic analysis revealed that CmPI belonged to the class B GLOBOSA/PISTILLATA subfamily of MADS TFs and was closely related to the *C. lavandulifolium* PI protein (Fig. S11a). Through multiple sequence alignment, we found that CmPI contained highly conserved domains that were typical of the MADS TFs (Fig. S11b). qRT-PCR showed that *CmPI* expression was higher in ray florets of “Dong Li Fen Gui” than in disc florets, similar to the expression pattern of *CmAP3* (Fig. 5a; Fig. S3). However, unlike CmAP3 and CmUIF1, CmPI was not able to interact directly with the *CmCCD4a-2* promoter (Fig. 5b). Y2H assays showed that yeast cells co-transformed with CmPI and CmAP3 constructs or CmPI and CmUIF1 constructs grew well on selective medium supplemented with 3AT and X-α-gal and turned blue (Fig. 5c), suggesting that CmPI could interact with CmAP3 and CmUIF1 separately. BiFC assays verified these results (Fig. 5d). We concluded that CmPI could be an intermediate “bridge” protein connecting CmAP3 and CmUIF1 in chrysanthemum.

### The CmAP3-CmPI-CmUIF1 TF complex activates the promoter of *CmCCD4a-2*

**Figure 6:**
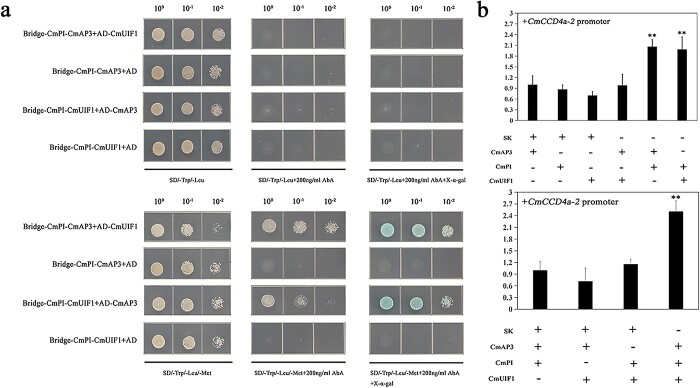
Effect of the CmAP3-CmPI-CmUIF1 TF complex on the activity of the *CmCCD4a-2* promoter. **a.** Y3H was carried out to detect the interaction among CmAP3, CmPI, and CmUIF1. **b.** A dual-luciferase assay showed that the CmAP3-CmPI-CmUIF1 heterotrimer effectively activated the promoter of *CmCCD4a-2*. All data are presented as the mean ± SD from at least three biological replicates (^*^P < 0.05; ^**^P < 0.01; Student’s *t*-test).

A Y3H assay was used to further verify the interaction between CmAP3, CmPI, and CmUIF1. Yeast cells containing both Bridge-CmPI-CmUIF1 and AD-CmAP3 constructs and Bridge-CmPI-CmAP3 and AD-CmUIF1 constructs grew well on SD/−Trp/−Leu/−Met medium with 200 ng/ml AbA (SD/−Trp/−Leu/−Met+200 ng/ml AbA), whereas they did not grow on SD/−Trp/−Leu medium with 200 ng/ml AbA (SD/−Trp/−Leu/+200 ng/ml AbA) (Fig. 6a), indicating that CmAP3 could interact with CmUIF1 indirectly only if CmPI was present. CmPI was therefore regarded as a physical “bridge” that connected CmAP3 and CmUIF1 to form the CmAP3-CmPI-CmUIF1 TF complex.

**Figure 7:**
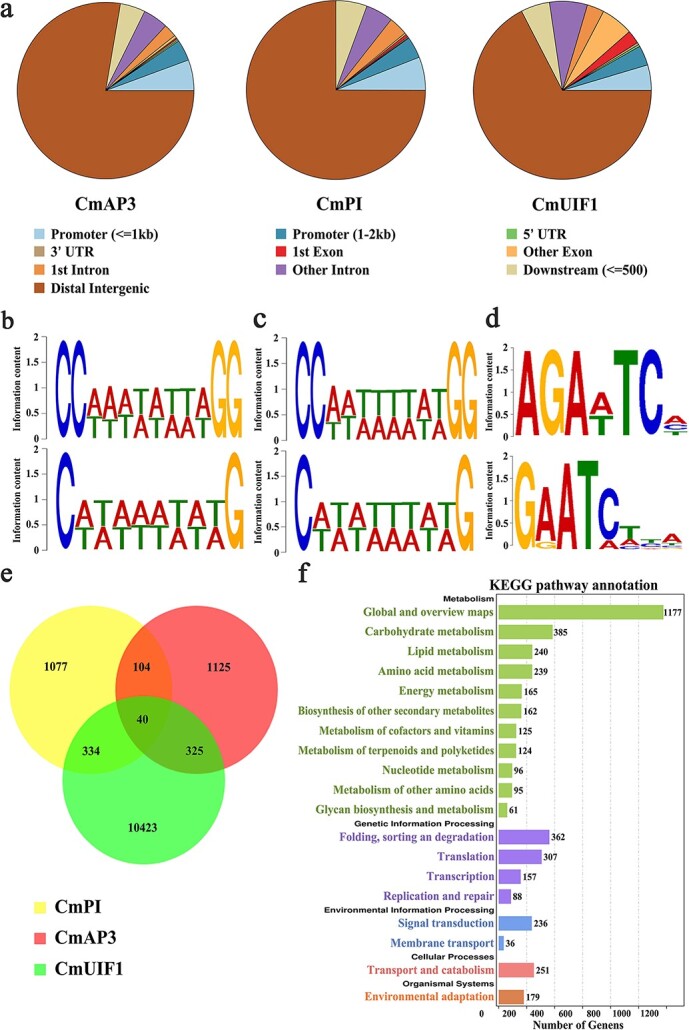
Downstream target genes regulated by the CmAP3-CmPI-CmUIF1 TF complex were identified by DAP-seq. **a.** Genome-wide analysis of the CmAP3/CmPI/CmUIF1-binding peaks. Conserved motifs of CmAP3 (**b**), CmPI (**c**), and CmUIF1 (**d**) identified from the DAP-seq data. **e.** Venn diagram showing peak overlap between the putative target genes related to CmAP3, CmPI, and CmUIF1-binding peaks. **f.** KEGG enrichment analysis of all putative target genes regulated by the CmAP3-CmPI-CmUIF1 TF complex.

Dual luciferase assays were conducted to investigate the effect of the CmAP3-CmPI-CmUIF1 TF complex on the activity of the *CmCCD4a-2* promoter. Relative luciferase expression (LUC/REN ratio) was significantly higher in the presence of the CmAP3-CmPI-CmUIF1 TF complex than in the control (Fig. 6b), indicating that the CmAP3-CmPI-CmUIF1 heterotrimer could effectively activate the promoter of *CmCCD4a-2* in chrysanthemum. We therefore proposed that the CmAP3-CmPI-CmUIF1 TF complex was abundant in the ray florets of the anemone-type chrysanthemum cultivar “Dong Li Fen Gui” and could bind directly to the *CmCCD4a-2* promoter and activate its transcription*.* This resulted in the accumulation of only trace amounts of carotenoids in the ray florets*.*

### Genome-wide binding site analysis of the CmAP3-CmPI-CmUIF1 TF complex

TF complexes usually modulate plant growth and development, fruit ripening, and secondary metabolism by controlling the transcription levels of multiple downstream structural or regulatory genes simultaneously. We used DNA affinity purification sequencing (DAP-seq), an *in vitro* DNA-TF binding assay that has been widely used to identify downstream target genes in plants [44, 45], to investigate putative downstream target genes regulated by the CmAP3-CmPI-CmUIF1 TF complex. First, recombinantly expressed CmAP3, CmPI, and CmUIF1 were incubated with gDNA libraries of *C. nankingense*, one of the key progenitors of domesticated chrysanthemum, and CmAP3/CmPI/CmUIF1-binding DNA fragments were enriched and sequenced using next-generation sequencing. A total of 4734/4945/43 115 (CmAP3/CmPI/CmUIF1) peaks were obtained, with a total length of 1 027 630/988 744/13 662 116 bp and an average length of 217/199/316 bp. We analyzed the distribution of these peaks located in annotated genic regions and found that 78.31%/77.37%/67.41% (CmAP3/CmPI/CmUIF1) were enriched in intergenic regions, 8.91%/8.88%/8.23% within gene promoter regions (2 kb upstream of the TSS), 4.14%/4.85%/5.26% within the 0.5-kb downstream regions, 1.63%/1.40%/8.33% within exon regions, and 6.55%/7.14%/9.98% within intron regions (Fig. 7a). To identify the DNA motifs of CmAP3, CmPI, and CmUIF1, we used MEME-ChIP and performed *de novo* motif discovery in the sequences of the DAP-seq peaks. CArG boxes, including C(A/T)_8_G and CC(A/T)_6_GG, were most strongly enriched for both CmAP3 and CmPI (Fig. 7b-c), consistent with previous reports [42, 43]. Significant putative DNA binding motifs AGA(A/T)TC(A/C/T), which were quite similar to that of AtUIF1 [23], were identified for CmUIF1 (Fig. 7d).

The putative target genes related to CmAP3/CmPI/CmUIF1-binding peaks were integrated, and a total of 13 428 genes were eventually identified (Fig. 7e). KEGG analysis was performed to further understand the biological functions of these target genes (Fig. 7f). *PSY1* (CHR00034017), *PSY2* (CHR00091590), *CRTISO* (CHR00019807), *LCYB* (CHR00027913), *CHYE* (CHR00027078), *VDE* (CHR00000429), and *ZEP* (CHR00026800), enriched in the carotenoid biosynthesis pathway (ko00906), were identified (Fig. S12a; Table S4). This result indicated that the CmAP3-CmPI-CmUIF1 TF complex might be involved in carotenoid biosynthesis in chrysanthemum. *CHS1* (CHR00038317), *CHS2* (CHR00047213), *CHS3* (CHR00008801), *CHI* (CHR00036904), *F3H* (CHR00037184), *FLS* (CHR00000448), *F3′H* (CHR00050525), and *DFR* (CHR00058078), which participate in flavonoid biosynthesis, were also directly bound by the CmAP3-CmPI-CmUIF1 TF complex (Fig. S12b; Table S4). We also found that this TF complex may be involved in chrysanthemum petal development by directly binding to flower symmetry *CYC2*-like genes such as *CYC2a* (CHR00035692, CHR00048990), *CYC2b* (CHR00065022), *CYC2d* (CHR00093761), and *CYC2f* (CHR00074698) (Fig. S13; Table S4).

## Discussion

### *CmCCD4a-2* is the most important carotenoid metabolic gene responsible for differences in carotenoid content of ray and disc florets

Carotenoid biosynthetic genes in plants have been studied for more than 30 years, but the role of carotenoid degradation has only been addressed more recently [46]. Carotenoids are cleaved by carotenoid cleavage dioxygenases (CCDs), and these enzymes are generically divided into five subfamilies: NCED (nine-cis-epoxycarotenoid dioxygenase), CCD1, CCD4, CCD7, and CCD8 [47]. A large number of studies have reported that the differential expression of *CCD1s* and *CCD4s* results in differences in carotenoid content in various plant species and cultivars, such as the different colored fruits of peach (*PpCCD4* [8,48]) and summer squash (*CpCCD4a* and *CpCCD4b* [49]) and the different colored flowers of chrysanthemum (*CmCCD4a* [9]), osmanthus (*OfCCD4* [10]), oncidium (*OgCCD1* [50]), and daffodil (*NpCCD4* [51]).

Our previous study found that *CmCCD4a-2*, which had high homology to *CmCCD4a*, was accompanied by *CmLCYE* and *CmPAPs* to simultaneously affect carotenoid accumulation in the ray florets of chrysanthemum cultivars with different colors [27]. Here, we found that of all the carotenoid metabolic genes, *CmCCD4a-2* was the only gene that was highly expressed in the ray florets of “Dong Li Fen Gui” at all developmental stages, and it had almost no expression in the disc florets, showing a negative correlation with carotenoid accumulation (Fig. 2c; Fig. 3a-b; Fig. S3). *CmCCD4a-2* was also highly expressed in the ray florets of other white- and pink-colored chrysanthemum cultivars, whereas only low transcript levels of *CmCCD4a-2* were detected in the disc florets, which accumulated a large amount of carotenoids (Fig. S5a-c; Fig. S3). These results indicated that the expression pattern of *CmCCD4a-2* was conserved and consistent in different colored chrysanthemum cultivars. Thus, we proposed that significantly different expression levels of *CmCCD4a-2* resulted in distinct carotenoid contents in ray and disc florets of chrysanthemum. However, it was not clear why *CmCCD4a-2* was differentially expressed in these two floret types.

Trans-acting factors, particularly upstream TFs, affect gene transcription levels by binding to cis-acting regulatory regions. Currently, only a few TFs have been found to directly regulate the expression of *CCD4*. OfWRKY3 and OfERF61 in osmanthus activate *OfCCD4* expression, resulting in carotenoid cleavage in petals [52, 53]. VvMADS4 in grapes negatively regulated the expression of *VvCCD4b* [54]. However, until now, the underlying mechanism by which TFs or TF complexes transcriptionally regulate *CmCCD4a-2* expression in chrysanthemum was poorly understood.

### The CmAP3-CmPI-CmUIF1 TF complex modulates carotenoid metabolism by directly regulating the expression of *CmCCD4a-2*

Through Y1H screening and Y1H assays, we first demonstrated that CmAP3 and CmUIF1 TFs could directly bind to the *CmCCD4a-2* promoter to perform regulatory functions (Fig. 4c; Table S3). Phylogenetic analysis revealed that CmAP3 belonged to the class B DEFICIENS/APETALA3 subfamily of the MADS-box TF family, and CmUIF1 was a member of the GARP TF family (Fig. S8a-b). Currently, many MADS TFs that directly regulate carotenoid metabolism have been characterized [6]. For example, RIN can affect carotenoid biosynthesis by directly regulating the transcription levels of several carotenoid metabolic genes during tomato fruit ripening [55, 56]. Tomato FUL1 has also been shown to promote the expression of *SlPSY1* by binding directly to its promoter [17, 18]. In citrus, CsMADS6 modulated the transcription levels of *LCYB1* together with *PSY* and *PDS* to affect carotenoid accumulation [57]. Members of the GARP TF family have been widely reported to participate mainly in hormonal signaling [58], nutrient response and sensing processes [59], chloroplast biogenesis [60], and plant development, particularly in leaves and flowers [23], but few studies have reported the regulation of carotenoid metabolism by GARP TFs.

**Figure 8:**
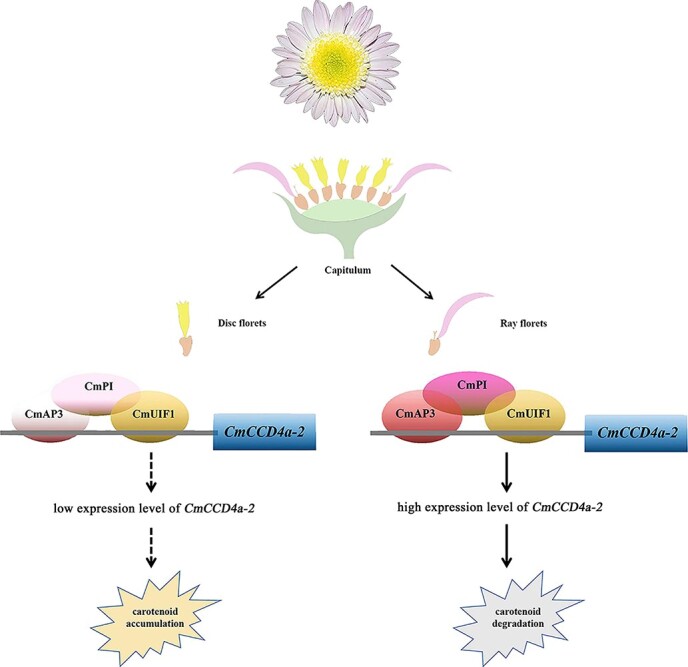
A regulatory model of differential carotenoid accumulation in ray and disc florets of chrysanthemum. The CmAP3-CmPI-CmUIF1 TF complex is abundant in the ray florets and can bind directly to the *CmCCD4a-2* promoter and effectively activate its transcription, resulting in the accumulation of only trace or low amounts of carotenoids. Low transcript abundance of *CmAP3* and *CmPI*, important components of the CmAP3-CmPI-CmUIF1 TF complex, was detected in the disc florets. There was not enough TF complex to effectively activate the expression of *CmCCD4a-2,* and this led to the accumulation of large amounts of carotenoids*.* The color saturation of the circles (deep/light) represents the expression levels of *CmAP3*, *CmPI*, and *CmUIF1* (high/low).

Dual luciferase assays were performed to test the effect of CmAP3 and CmUIF1 on the activity of the *CmCCD4a-2* promoter. However, the results showed that the C*mCCD4a-2* promoter was not activated when CmAP3 or CmUIF1 was present alone (Fig. 4d). TFs usually regulate secondary metabolism, including carotenoid metabolism, by forming TF complexes. For example, WHITE PETAL1 (MtWP1) in *Medicago truncatula* physically interacts with MtTT8 and MtWD40–1 to promote carotenoid-derived flower pigmentation [14]. CsMADS5, a positive regulator, interacts with CsMADS6 to synergistically promote carotenoid accumulation [19]. Therefore, we analyzed the relationships among CmAP3, CmUIF1, and CmPI, which belongs to the class B GLOBOSA/PISTILLATA subfamily of MADS-box TFs (Fig. S11) and has been reported to interact with AP3 to determine the organ identity of petals and stamens [21, 61]. The results showed that CmAP3 was not able to interact with CmUIF1 (Fig. S10a-b), whereas CmPI, regarded as a physical “bridge”, could connect CmAP3 and CmUIF1 to form the CmAP3-CmPI-CmUIF1 TF complex in chrysanthemum (Fig. 5c-d; Fig. 6a). Dual luciferase assays showed that the CmAP3-CmPI-CmUIF1 TF complex could effectively activate the promoter of *CmCCD4a-2* (Fig. 6b)*.* In summary, we proposed a working model: the CmAP3-CmPI-CmUIF1 TF complex is abundant in the ray florets of the anemone-type chrysanthemum cultivar “Dong Li Fen Gui” and can bind directly to the *CmCCD4a-2* promoter and effectively activate its transcription, resulting in the accumulation of only trace or low amounts of carotenoids. A low abundance of transcripts of *CmAP3* and *CmPI*, important components of the CmAP3-CmPI-CmUIF1 TF complex, was detected in the disc florets of “Dong Li Fen Gui” (Fig. 4b; Fig. 5a). There was not enough TF complex in the disc florets to effectively activate the expression of *CmCCD4a-2,* and this promoted the abundant accumulation of carotenoids (Fig. 8)*.*

### The CmAP3-CmPI-CmUIF1 TF complex may participate in multiple processes in chrysanthemum

A number of TFs or TF complexes that simultaneously regulate multiple secondary metabolic processes have been characterized [14, 15, 62]. For example, AdMYB7, an R2R3-MYB TF in kiwifruit, regulated carotenoid accumulation via the transcriptional activation of the *AdLCYB* promoter. Overexpression of the *AdMYB7* gene in *N. benthamiana* altered chlorophyll accumulation and increased the expression of the chlorophyll biosynthesis genes *NbGGR* and *NbSGR1* [15]. WHITE PETAL1 (MtWP1) in *M. truncatula* interacted with MtTT8 and MtWD40-1 to activate the expression of both carotenoid and anthocyanin biosynthetic genes [14]. SlMYB72 in tomato interacted with the auxin response factor SlARF4 to regulate chlorophyll, carotenoid, and flavonoid biosynthesis simultaneously [62]. In this study, a large number of metabolic genes directly bound by the CmAP3-CmPI-CmUIF1 TF complex, including carotenoid biosynthetic genes such as *PSY1, PSY2, CRTISO, LCYB, CHYE, VDE,* and *ZEP* and flavonoid biosynthetic genes such as *CHS1*, *CHS2*, *CHS3*, *CHI*, *F3H*, *FLS*, *F3′H*, and *DFR*, were identified through DAP-seq (Fig. S12a-b; Fig. S13; Table S4). These results indicated that the CmAP3-CmPI-CmUIF1 TF complex might be involved not only in carotenoid degradation (as mentioned above, this TF complex could directly regulate the expression of *CmCCD4a-2,* one of the most important structural genes in carotenoid degradation) but also in the biosynthesis of carotenoids and flavonoids. However, the specific regulatory mechanism requires further investigation.

Some transcription factors from the CYCLOIDEA/TEOSINTE BRANCHED1 (CYC/TB1) or MADS-box TF families that were reported to participate in pigment metabolism still play a very important role in flower and fruit development [57, 63]. In *Torenia fournieri,* TfCYC2 bound directly to the regulatory regions of *TfMYB1* to establish an asymmetric pigmentation pattern. When *TfCYC2* was up- or downregulated, dorsal petal identity, petal shape, and corolla pigmentation pattern were simultaneously changed [63]. CsMADS6 in citrus activated the transcription of *CsLCYB1* along with *CsPSY*, *CsPDS,* and *CsCCD1* to modulate carotenoid metabolism. Overexpression of *CsMADS6* in tomato dramatically altered carotenoid accumulation, and this was accompanied by changes in sepal morphology [57]. Here, some flower development-related genes, particularly flower symmetry *CYC2*-like genes, such as *CYC2a* (CHR00035692, CHR00048990), *CYC2b* (CHR00065022), *CYC2d* (CHR00093761), and *CYC2f* (CHR00074698) (Fig. S13; Table S4), were identified and appeared to be potential target genes regulated by the CmAP3-CmPI-CmUIF1 TF complex based on DAP-seq data. Developmental genetic studies have found that *CYC2*-like genes are the key genes that participate in the regulation of organ symmetry in many eudicot plant groups [64, 65]. These *CYC2*-like members are also involved in capitulum architecture, allometric growth and fusion of corolla lobes, and stamen regression in Asteraceae [66–68]. Among them, *CYC2b* and *CYC2e* were more likely to participate in regulating the petal ligule length of ray florets [66, 69]. *CYC2c* and *CYC2g* determined the position and zygomorphy of ray florets [68]. *CYC2d* was shown to repress the growth of stamens and dorsal corolla lobes [61, 62]. In general, research on the regulatory mechanism of *CYC2*-like genes has focused mainly on protein–protein interactions [70, 71], whereas the regulatory networks upstream of these genes are poorly understood. Our data showed that the CmAP3-CmPI-CmUIF1 TF complex may serve as a potential upstream TF complex that binds directly to the regulatory regions of *CYC2*-like genes to affect the development of ray and disc florets in chrysanthemum. However, the specific regulatory mechanism requires further analysis.

## Supplementary Material

Web_Material_uhac020

## Data Availability

All data supporting the findings of this study are available within the paper and within its supplementary materials published online.
