# Supplementary material for: The transcription factor complex CmAP3-CmPI-CmUIF1 modulates carotenoid metabolism by directly regulating the carotenogenic gene CmCCD4a-2 in chrysanthemum
Source: Hortic Res. 2022 Feb 19;9:uhac020. doi: 10.1093/hr/uhac020 (PMC9125392; doi:10.1093/hr/uhac020)
Supplement: Web_Material_uhac020 [file web_material_uhac020.doc]

**Supplementary figures and tables**

**
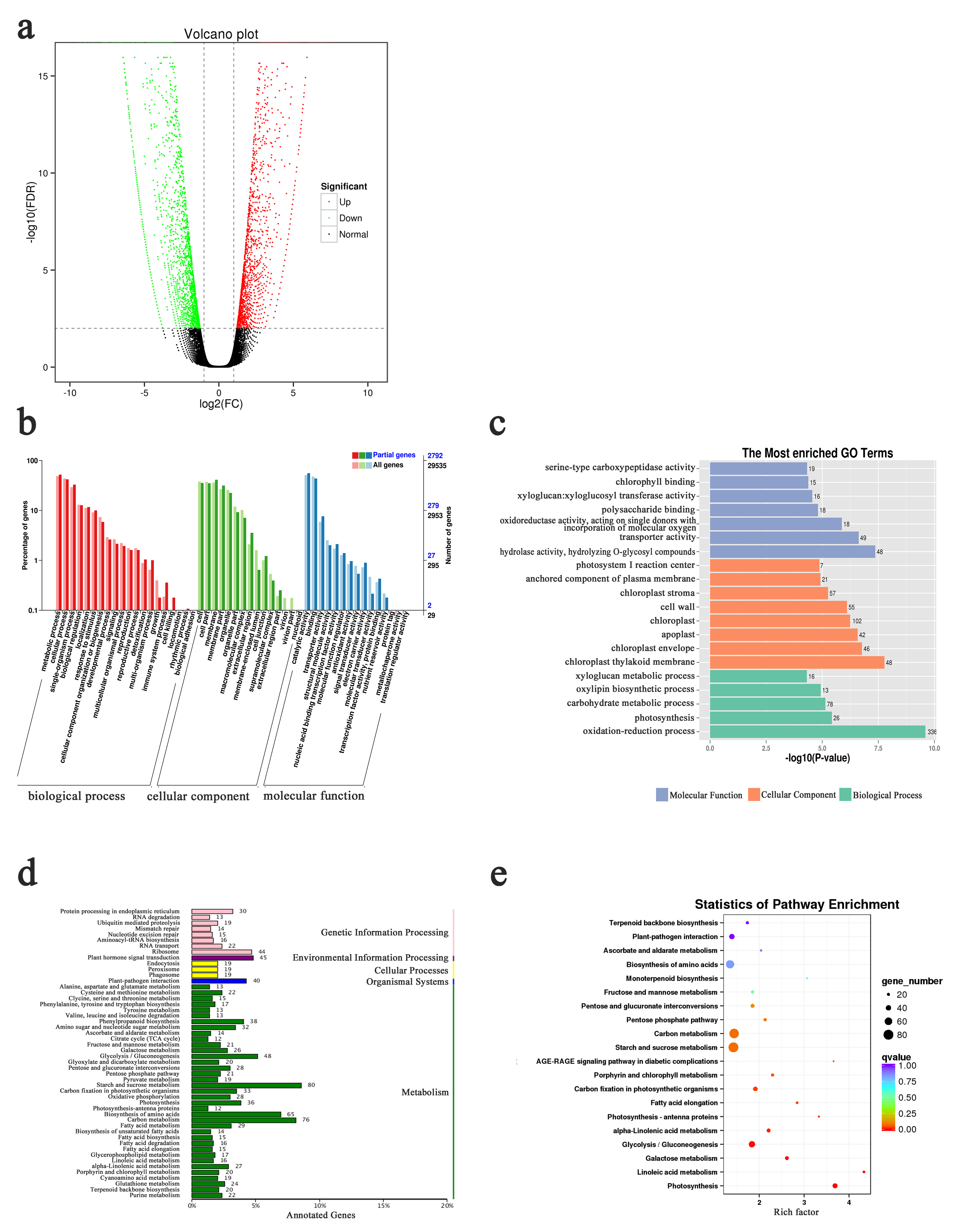
**

**Fig. S1 Screening of differential expression genes (DEGs) and GO and KEGG pathway enrichment analysis. a.** The volcano plot showing the DEGs in the ray and disc florets. **b.** Functional classification of differential expression genes by GO categorization. **c.** GO terms enrichment analysis. **d.** Pathway assignment by KEGG classification. **e.** KEGG analysis of the DEGs.


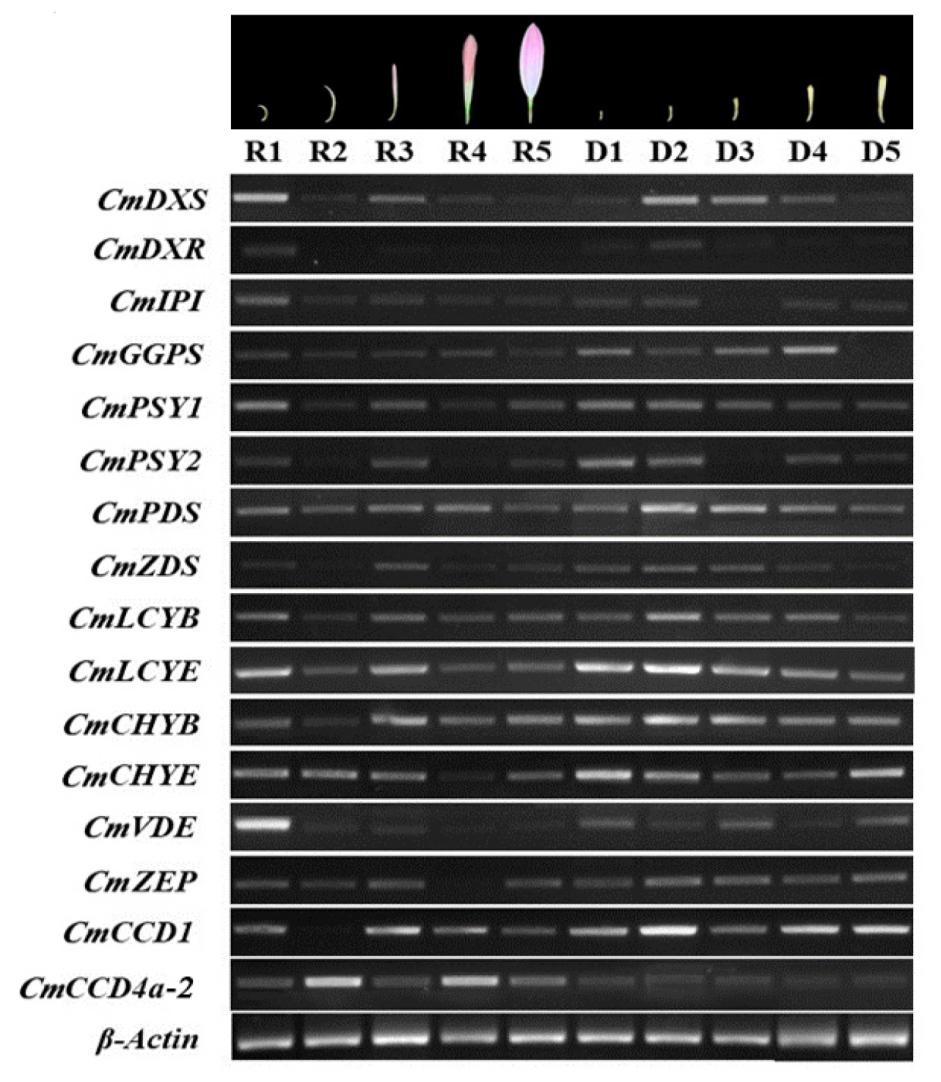


**Fig. S2 Expressed pattern of metabolic genes in ray and disc florets of ‘Dong Li Gui Gen’ at different developmental stages of capitulum (S1-S5) by** semi-quantitative **RT-PCR.** The transcripts of reference gene *β-actin* was used to normalize the cDNA levels of each experimental sample.


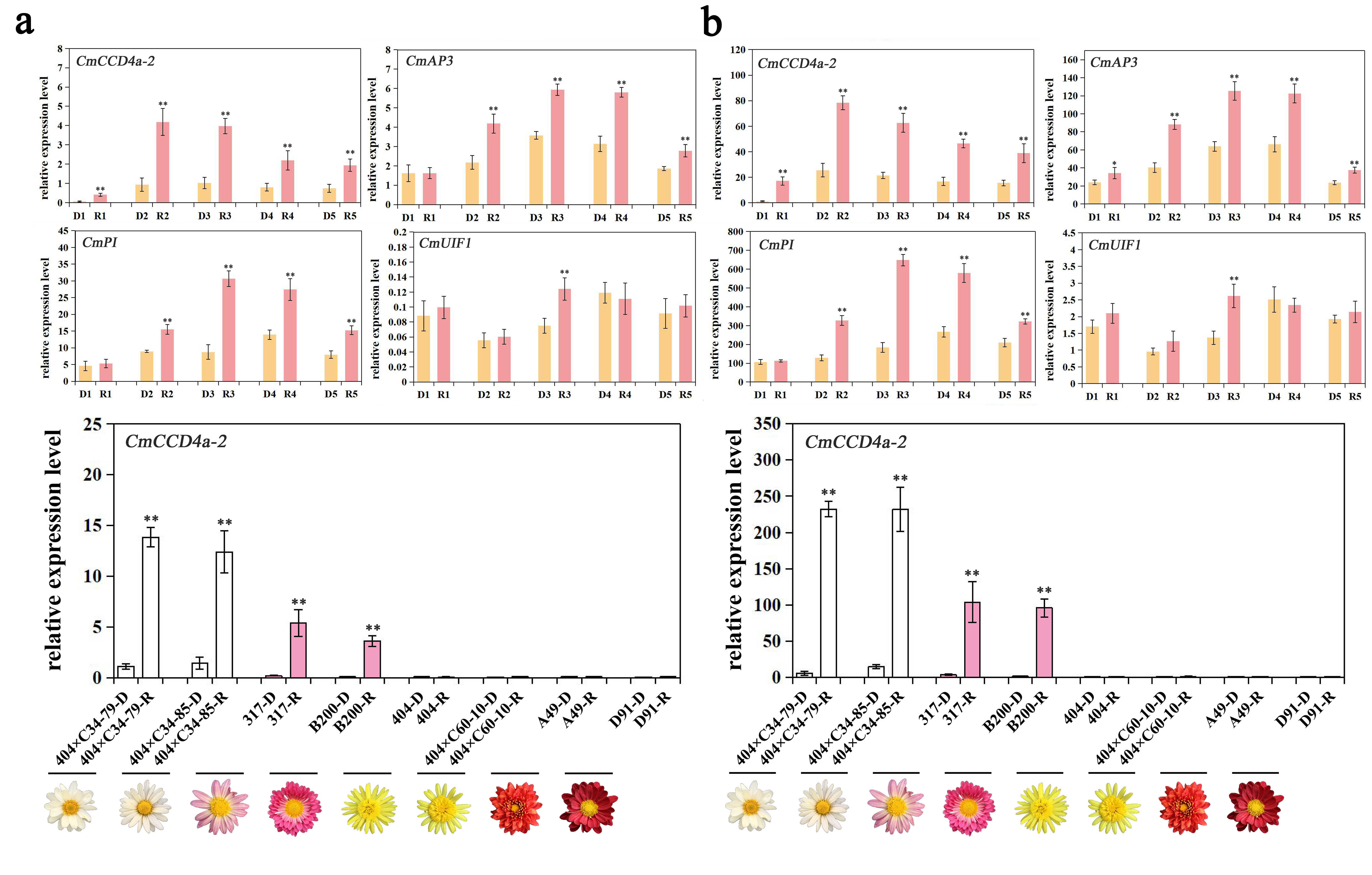


**Fig. S3 The expression patterns of *CmCCD4a-2, CmAP3, CmPI* and *CmUIF1* in the ray and disc florets were analyzed by qRT-PCR using *SAND* (a) and *F-box* (b) as new reference genes for normalization.** All data were presented as the mean ± SD from at least three biological replicates. (*, *P* < 0.05; **, *P* < 0.01; Student’s *t*-test).


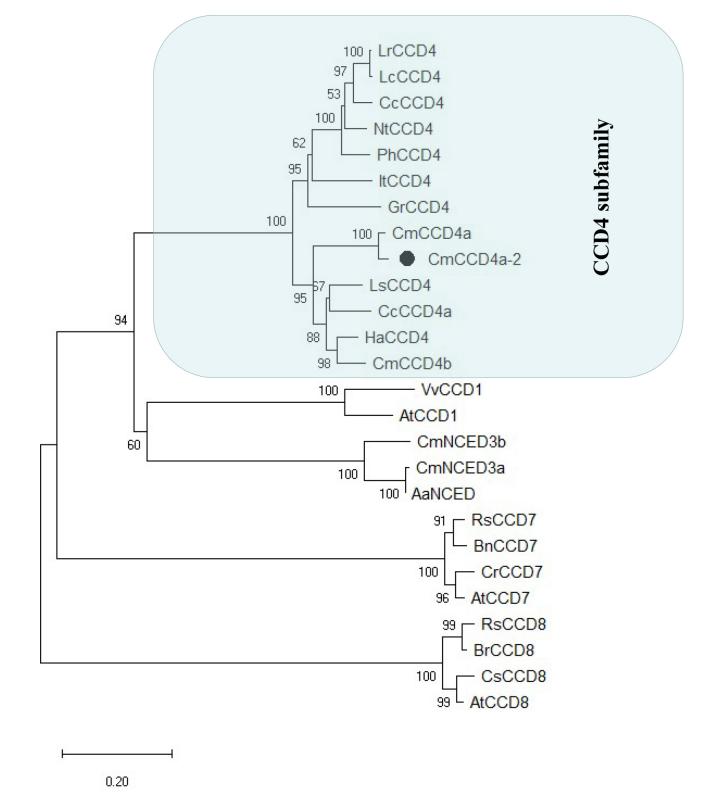


**Fig. S4 Phylogenetic tree of CmCCD4a-2 constructed.**


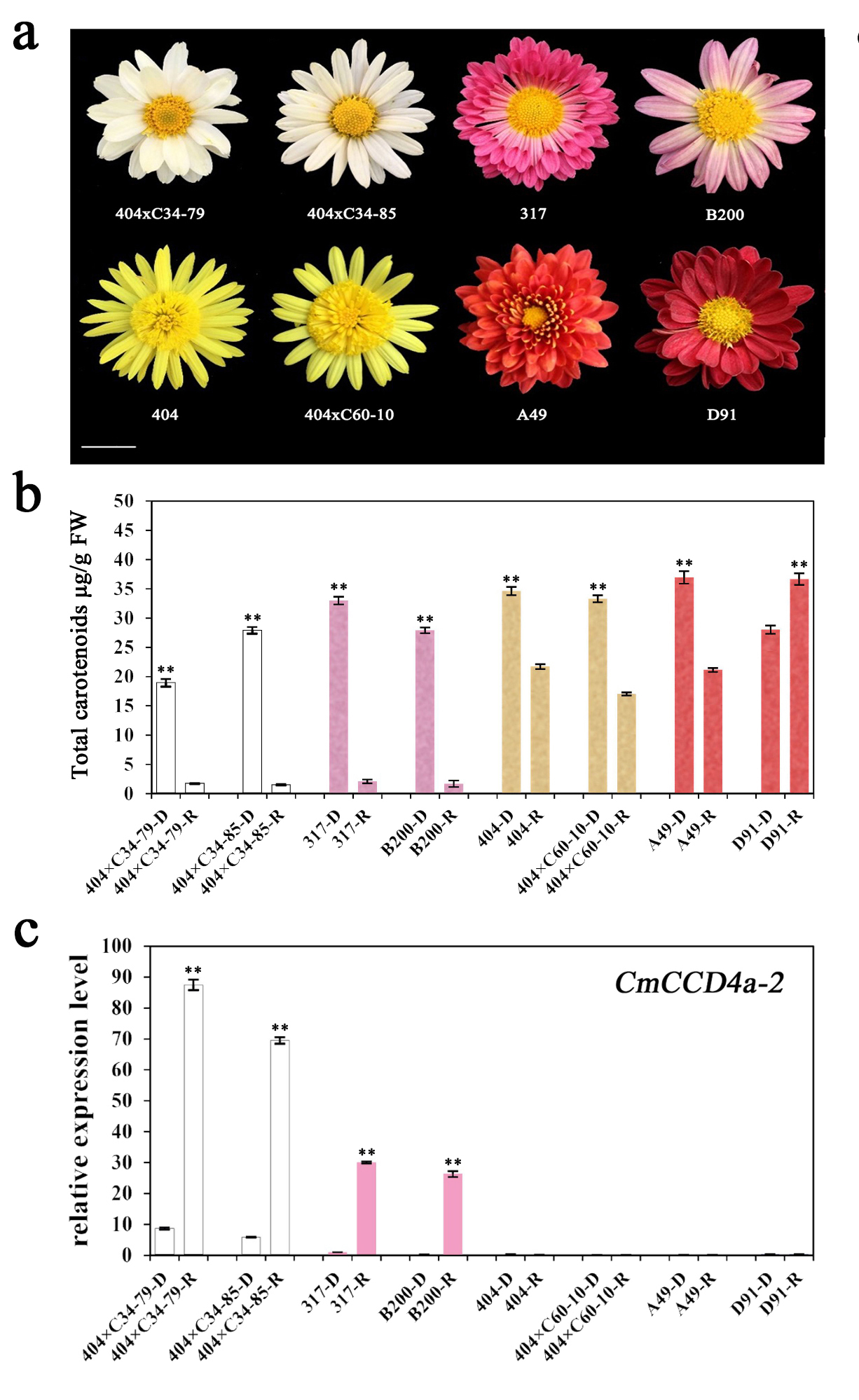


**Fig. S5 Differential expression of *CmCCD4a-2* caused the distinct content of carotenoids to accumulate in the ray and disc florets. a.** Characteristics of different colored chrysanthemum cultivars. Bar, 1 cm. **b.** Carotenoid contents analysis of ray and disc florets of different colored chrysanthemum cultivars. **c.** The expressed pattern of *CmCCD4a-2* in ray and disc florets was analyzed by qRT-PCR. All data were presented as the mean ± SD from at least three biological replicates. (*, *P* < 0.05; **, *P* < 0.01; Student’s *t*-test).


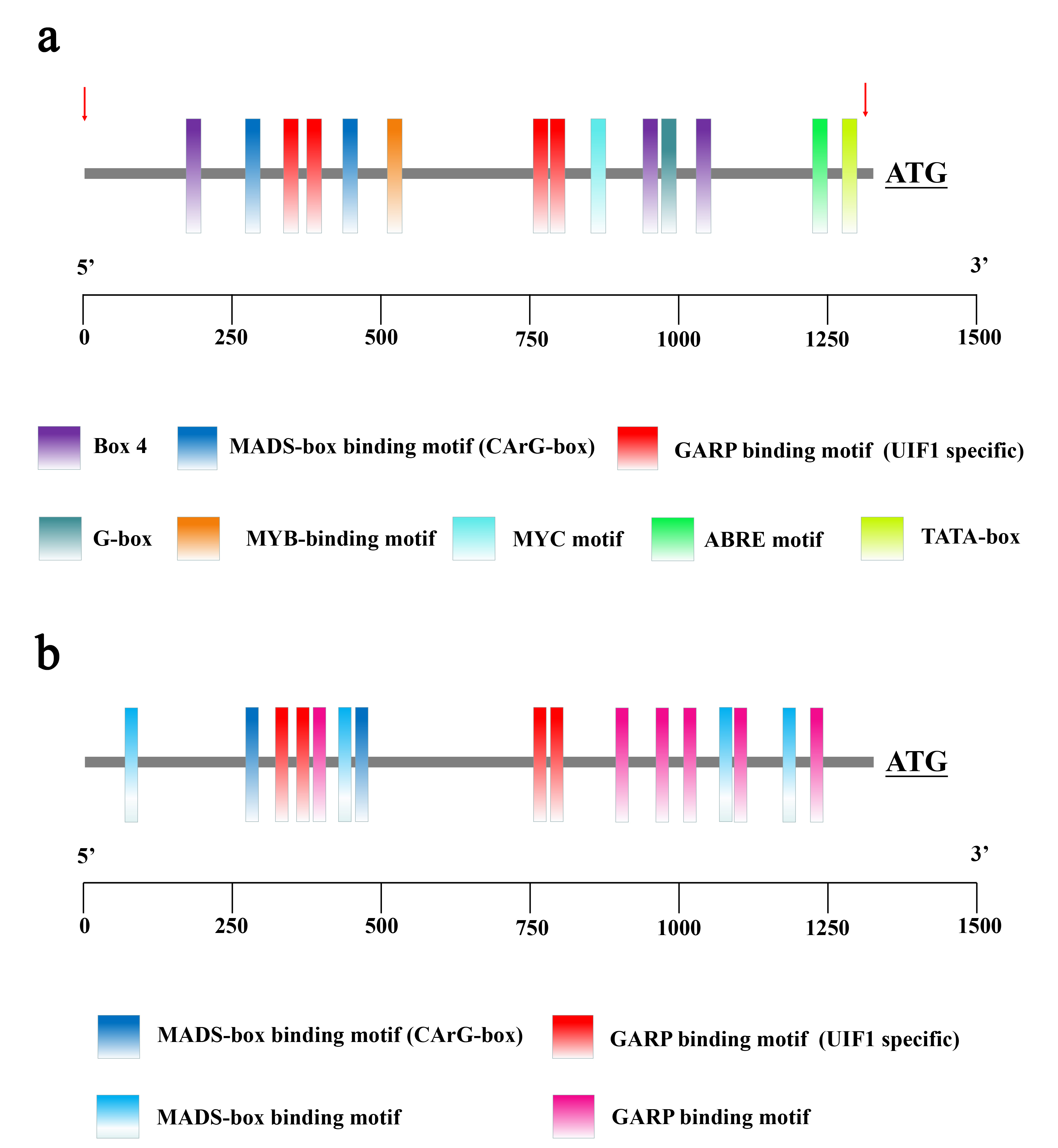


**Fig. S6 Cis-acting elements analysis of *CmCCD4a-2* promoter.** a**.** Possible cis-acting elements were predicted using PlantCare online database. b. Candidate MADS-box and GARP binding motifs were predicted by PlantPAN 3.0 online datebase.


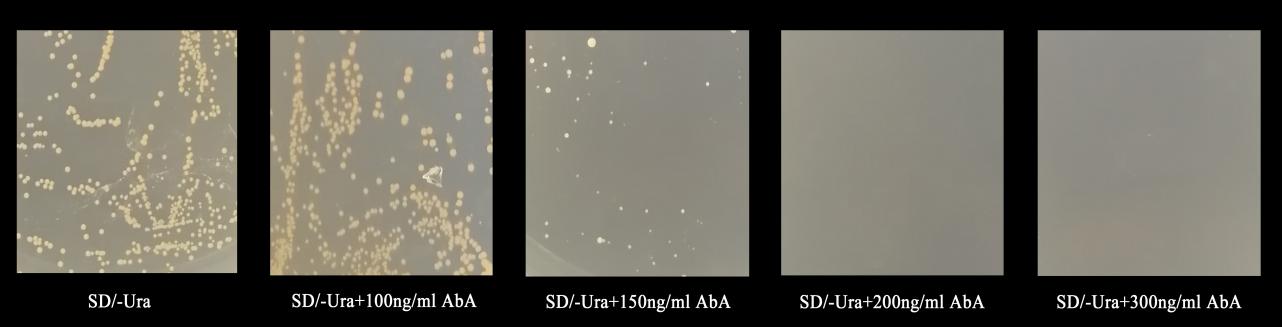


**Fig. S7 Growth of bait yeast strain integrating pAbAi-*CmCCD4a-2* promoter on the SD/-Ura and SD/-Ura+100/150/200/300 ng/ml Aureobasidin A (AbA) medium.**


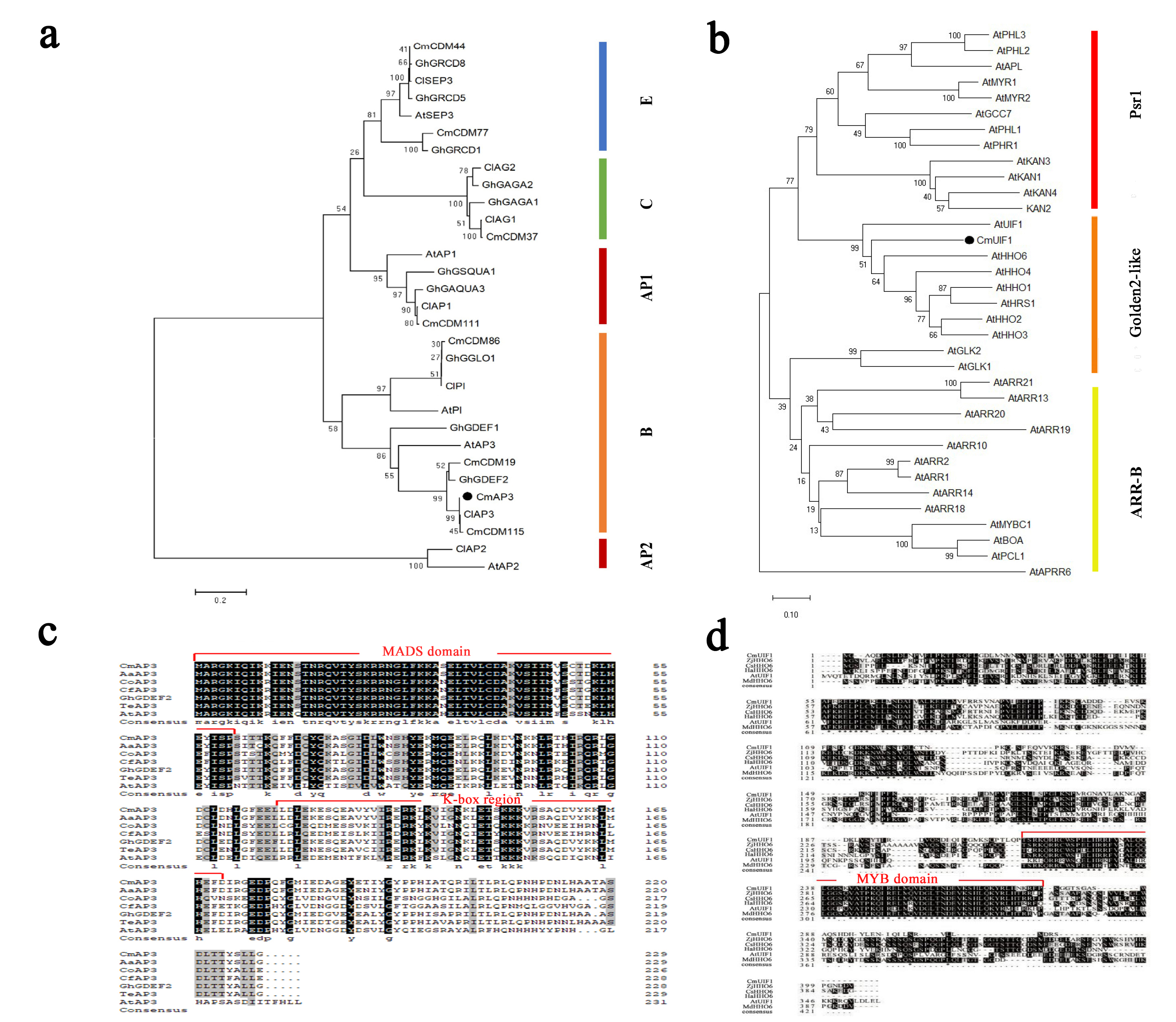
**Fig. S8 Sequence analysis of upstream transcription factors CmAP3 and CmUIF1. a.** Phylogenetic tree of CmAP3. **b.** Phylogenetic tree of CmUIF1. **c.** Sequence alignment of CmAP3 (*Chrysanthemum × morifolium*), AaAP3 (*Artemisia annua*), CoAP3 (*Camellia oleifera*), CfAP3 (*Cornus florida*), GhGDEF2 (*Gerbera hybrida*), TeAP3 (*Tagetes erecta*) and AtAP3 (*Arabidopsis thaliana*). The red line represented the conserved domains of the MADS-box TF family. **d.** Sequence alignment of CmUIF1 (*Chrysanthemum × morifolium*), ZjHHO6 (*Ziziphus jujuba*), CsHHO6 (*Camellia sinensis*), HaHHO6 (*Helianthus annuus*), MdHHO6 (*Malus domestica*), and AtUIF1 (*Arabidopsis thaliana*). The red line represented the conserved domain of the GARP TF family.

**
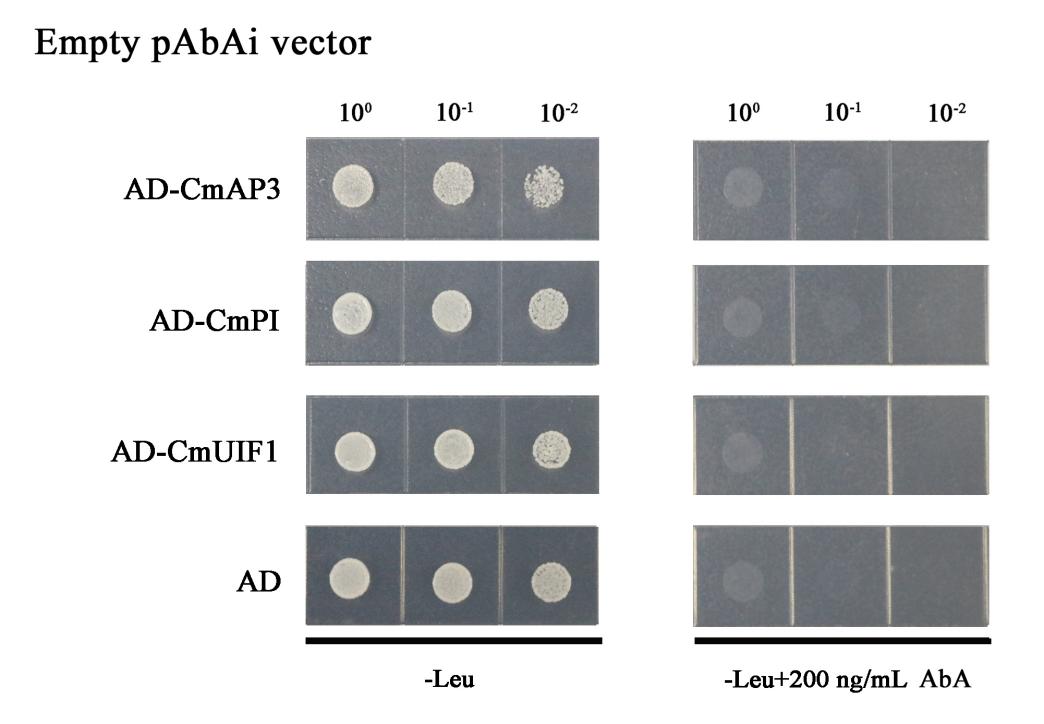
**

**Fig. S9 Interaction analysis between CmAP3/CmUIF1/CmPI and the minimal promoter region of pAbAi vector (as negative control ) by Y1H assay.**

**
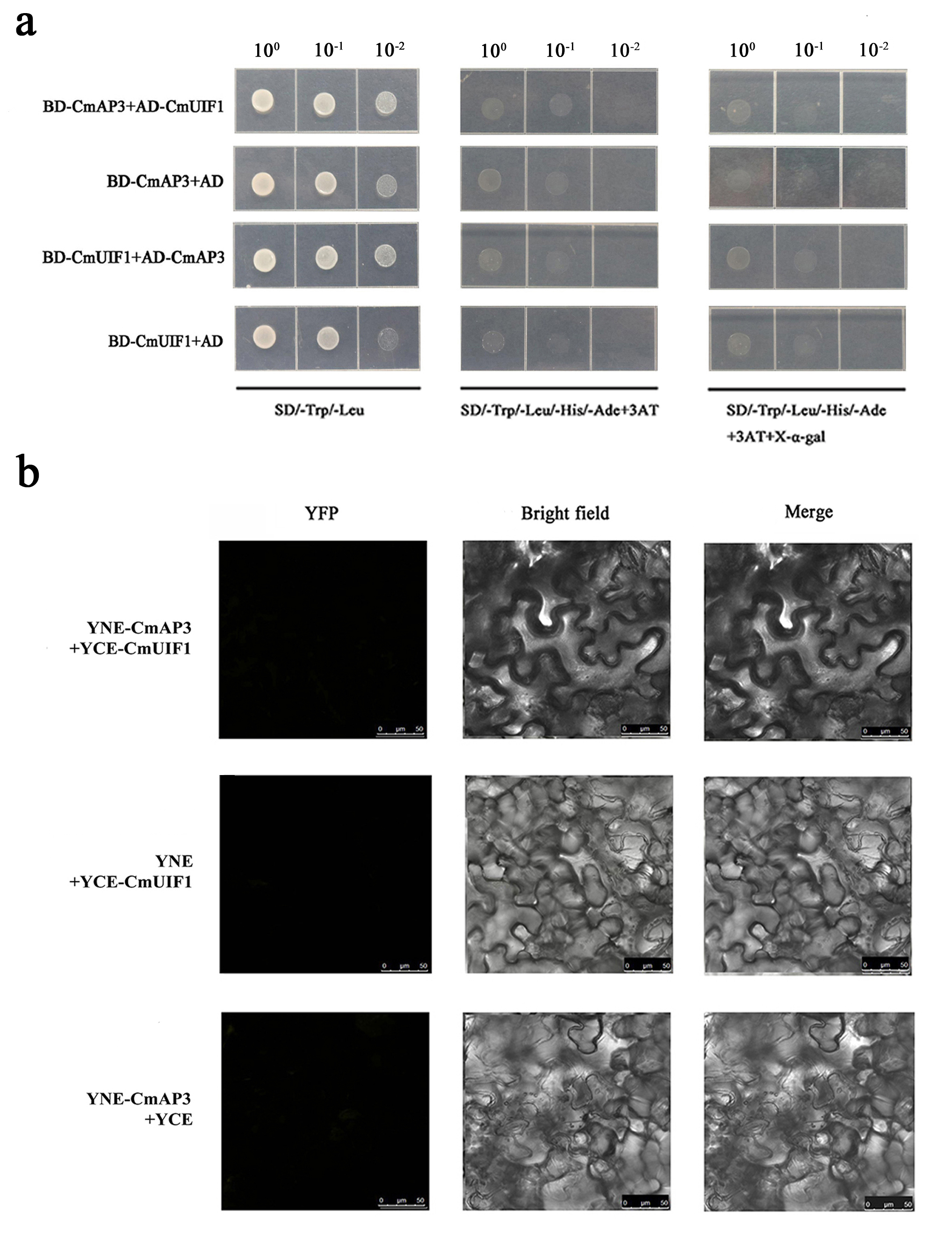
**

**Fig. S10 Protein-protein interaction between upstream transcription factors CmAP3 and CmUIF1.** a**.** Protein-protein interaction analysis between CmAP3 and CmUIF1 by Y2H assay. **b.** BiFC assay confirmed the interaction between CmAP3 and CmUIF1 in *Nicotiana benthamiana* leaves. YFP fluorescence signal was imaged 48 hours after injection by a laser scanning confocal microscope.


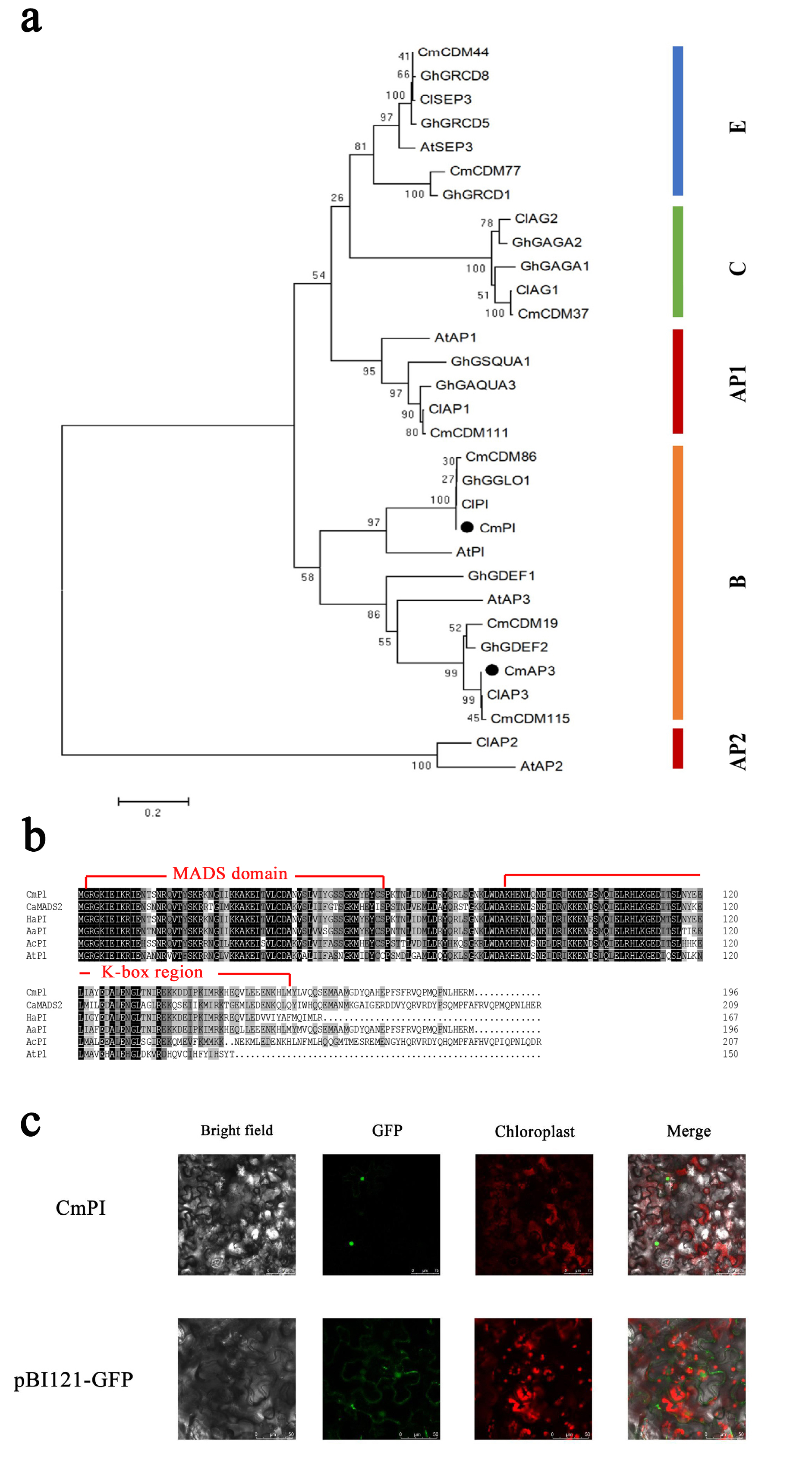


**Fig. S11 Sequence analysis and subcellular location of CmPI.** **a.** Phylogenetic tree of CmPI. **b.** Sequence alignment of CmPI (*Chrysanthemum × morifolium*), AaAP3 (*Artemisia annua*), CoAP3 (*Camellia oleifera*), CfAP3 (*Cornus florida*), GhGDEF2 (*Gerbera hybrida*), TeAP3 (*Tagetes erecta*) and AtAP3 (*Arabidopsis thaliana*). The red line represented the conserved domains of the MADS-box TF family. **c.** Subcellular localization of CmPI in tobacco leaves. The green GFP fluorescence signal was imaged 48 hours after injection by a laser scanning confocal microscope.


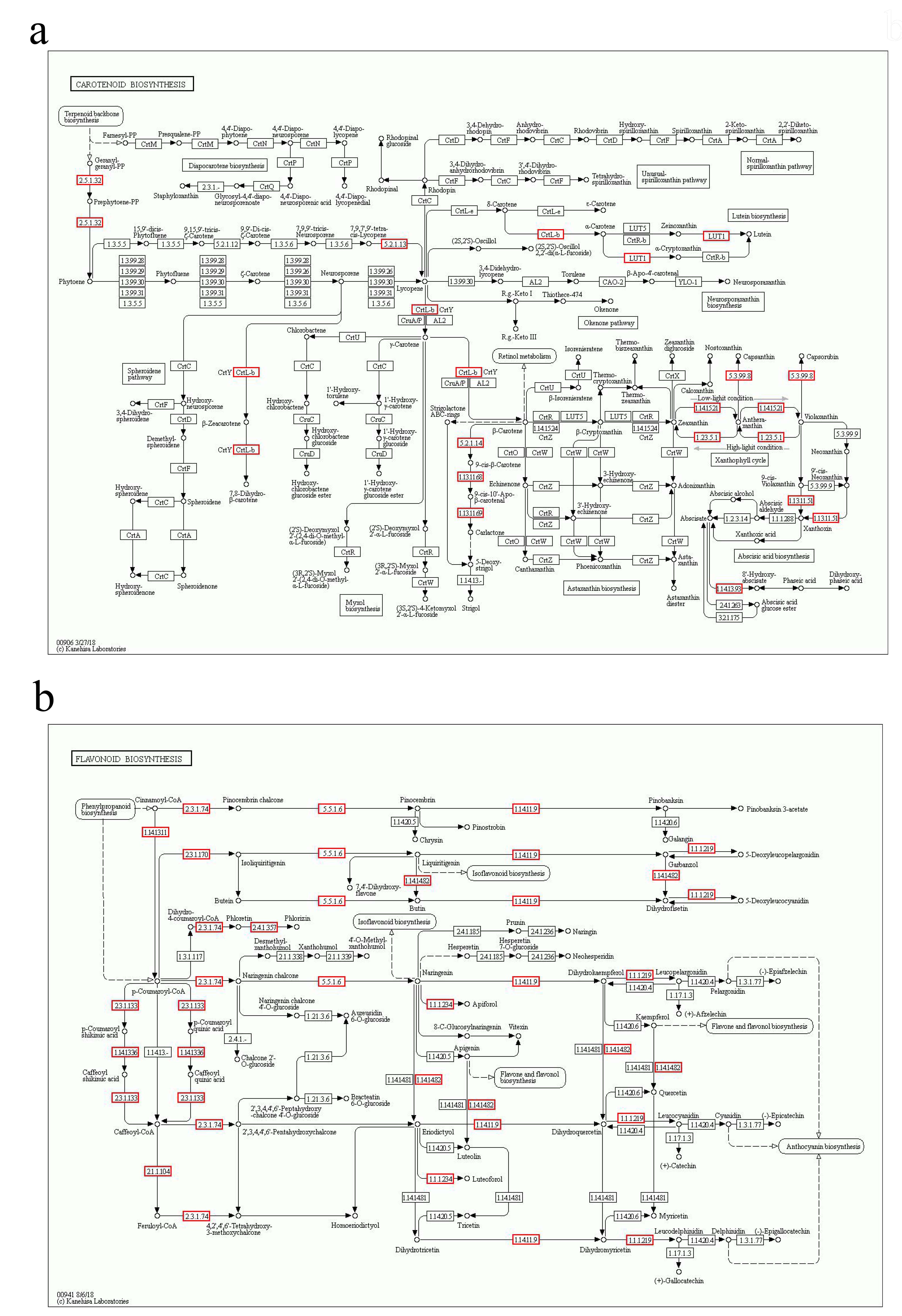


**Fig. S12 Candidate target genes enriched in carotenoid synthesis pathway (a) and flavonoid synthesis pathway (b) by KEGG pathway enrichment analysis.**





**Fig. S13 CmAP3-CmPI-CmUIF1 transcription factor complex might participate in multiple processes in chrysanthemum.** A large number of metabolic genes including carotenoid biosynthetic genes such as *PSY1, PSY2, CRTISO, LCYB, CHYE, VDE, ZEP* and flavonoid biosynthetic genes such as *CHS1*, *CHS2*, *CHS3*, *CHI*, *F3H*, *FLS*, *F3'H*, and *DFR*, as well as some flower development-related genes, particularly flower symmetry *CYC2*-like genes were directly bound by the CmAP3-CmPI-CmUIF1 TF complex based on DAP-seq data.

**Table S1 A list of primer sequences.**

| **Primers** | **Sequences（5’-3’）** | **Application** |
| --- | --- | --- |
| DXS-F | ATCAGGCATACCCACATAAG | semi-quantitative RT-PCR |
| DXS-R | CATACCCCTCGCATACTCA |
| DXR-F | GGTTCTATTGGGACTCAG |
| DXR-R | TCAGGGAAGCCTTGTAT |
| IPI-F | TCGCCACCGCAACAAAACT |
| IPI-R | TCACGCACGATGAAGAGCAG |
| GGPS-F | TTACCAGACCCGAAAATCAC |
| GGPS-R | AAACCGACATCAGCACCTT |
| PSY1-F | GTGGCTAATACAACAGGGAA |
| PSY1-R | GGCTGAATGTCAACAGGAAA |
| PSY2-F | ACTGTCTCCAAGTTCCC |
| PSY2-R | CACCGTTTCCATTTCCT |
| PDS-F | AATGTCTCCGCCTCTTTAG |
| PDS-R | TAGAATGCTCCTTCCACTG |
| ZDS-F | TGCTGTGGCTCTTGCTCTA |
| ZDS-R | TGATTGTCTGGCTCGTTCT |
| LCYB-F | GCGATGCCCTTTTCGTCTA |
| LCYB-R | AAACCGTCTTGTGCCTTCT |
| LCYE-F | GCGGCTTCGGGTAAACTTCT |
| LCYE-R | GTTTCCCACGCTTGCTTTGA |
| CHYB-F | GCCCACCTTGCCCAAAACT |
| CHYB-R | CCCTAAACCTGCCTTCCGTATC |
| CHYE-F | GTCAATGTTGGTTGCTGG |
| CHYE-R | TCGGTGTTGGCTTCGTTT |
| VDE-F | AAACCATCATCCCACAGC |
| VDE-R | GGTAAAGCACGCCCAAAA |
| ZEP-F | TGCTCTTACTGGCGAATGGTT |
| ZEP-R | TCTCCTCCCTCCTCGGTAATCT |
| CCD1-F | ACATTCACCGCTCACCCTA |
| CCD1-R | AACACCTCCACCGACCTTA |
| CCD4a-2-F | GGGCTCTTTTCCCACATCT |
| CCD4a-2-R | TGCTCCATCAAGGCAACTA |
| qRT-β-actin-F | TAAGAACGATAAGTGCCCACATAG | qRT-PCR |
| qRT-β-actin-R | TTTTAGACATCAGCCATAACAGAGT |
| qRT-SAND-F | CGTTGCTCACTACGAGTTCAC |
| qRT-SAND-R | GCAGATGGGTCAACAGGTAA |
| qRT-F-box-F | ACCAGAACCTGCTTTATGTATCGG |
| qRT-F-box-R | GAACGAAAACGAGAGGGGCT |
| qRT-CCD4a-2-F | GTCATTACCCCAGCCATCAG |
| qRT-CCD4a-2-R | TTTGTTCGGTGTTTACGAGTTG |
| qRT-AP3-F | ACCCACCACATATCGCTACACA |
| qRT-AP3-R | GCAAGGAATAGGTGGTGAGGTCT |
| qRT-PI-F | CATGAGCAAGTTTTGGAGGAGG |
| qRT-PI-R | CTGGACACGAAATGAAAAGGGT |
| qRT-UIF1-F | AAATGCCCCAGTGATTGAAGAG |
| qRT-UIF1-R | GGACTAATAAGCGAAAGCCCAGA |
| Luc-CCD4a-2-F | CTATAGGGCGAATTGGGTACCACCCTTCAACCCATTCATCTTCAAATT | Dual luciferase reporter assays |
| Luc-CCD4a-2-R | CGCTCTAGAACTAGTGGATCCAATTTGGTAAGGGATGCCTTACTACT |
| SK-CmAP3-F | CCGCGGTGGCGGCCGCTCTAGAATGGCTAGAGGAAAG |
| SK-CmAP3-R | TTCAGCGTACCGAATTGGTACCCTAGCCAAGCAAGGA |
| SK-CmPI-F | CCGCGGTGGCGGCCGCTCTAGAATGGGTAGGGGAAAGAT |
| SK-CmPI-R | TTCAGCGTACCGAATTGGTACCTCACATCCTCTCATGCA |
| SK-CmUIF1-F | CCGCGGTGGCGGCCGCTCTAGAATGGGTGCACAGGATT |
| SK-CmUIF1-R | TTCAGCGTACCGAATTGGTACCCTATACATAAGTTTCT |
| YCE-CmPI-F | GCTGGGCCCAGGCCTACTAGTATGGGTAGGGGAAAGAT | Bimolecular fluorescence complementation (BiFC) assays |
| YCE-CmPI-R | CTCCTACCCGGGAGCGGTACCTCACATCCTCTCATGCA |
| YCE-CmUIF1-F | GCTGGGCCCAGGCCTACTAGTATGGGTGCACAGGATT |
| YCE-CmUIF1-R | CTCCTACCCGGGAGCGGTACCCTATACATAAGTTTCT |
| YNE-CmAP3-F | TGGCGCGCCACTAGTGGATCCATGGCTAGAGGAAAG |
| YNE-CmAP3-R | CTCCATCCCGGGAGCGGTACCGCCAAGCAAGGA |
| YNE-CmUIF1-F | TGGCGCGCCACTAGTGGATCCATGGGTGCACAGGATT |
| YNE-CmUIF1-R | CTCCATCCCGGGAGCGGTACCTACATAAGTTTCT |
| BD-CmAP3-F | CATGGAGGCCGAATTCATGGCTAGAGGAAAG | Y1H, Y2H and Y3H assays |
| BD-CmAP3-R | GCAGGTCGACGGATCCCTAGCCAAGCAAGGA |
| BD-CmPI-F | CATGGAGGCCGAATTCATGGGTAGGGGAAAGAT |
| BD-CmPI-R | GCAGGTCGACGGATCCTCACATCCTCTCATGCA |
| BD-CmUIF1-F | CATGGAGGCCGAATTCATGGGTGCACAGGATT |
| BD-CmUIF1-R | GCAGGTCGACGGATCCCTATACATAAGTTTCT |
| AD-CmAP3-F | GCCATGGAGGCCAGTGAATTCATGAAGCGTGCTTGTAAT |
| AD-CmAP3-R | CAGCTCGAGCTCGATGGATCCTCATAGTTGCTCTGAAT |
| AD-CmPI-F | GCCATGGAGGCCAGTGAATTCATGAATGAAGCTTGTAGT |
| AD-CmPI-R | CAGCTCGAGCTCGATGGATCCTTATAGTTGCTCTGAATCT |
| AD-CmUIF1-F | GCCATGGAGGCCAGTGAATTCATGGGAAGGTCTCCTTGT |
| AD-CmUIF1-R | CAGCTCGAGCTCGATGGATCCTTATTTCATCTCCAAACCT |
| pAbAi-CCD4a-2-F | AAGCTTGAATTCGAGCTCACCCTTCAACCCATTCATCTTCAAATT |
| pAbAi-CCD4a-2-R | GAGCACATGCCTCGAGGTCGACAATTTGGTAAGGGATGCCTTACTACT |
| pGADT7-F | TAATACGACTCACTATAGGGC |  |
| pGADT7-R | AGATGGTGCACGATGCACAG |
| pBridge-CmPI-F | AAGAAGAGAAAGGTGGCGGCCGCATGGGTAGGGGAAAGAT |
| pBridge-CmPI-R | GGGAGATCAGCCCGAAGATCTTCACATCCTCTCATGCA |
| pBridge-CmPI-  CmAP3-F | TTGACTGTATCGCCGGAATTCATGGCTAGAG GAAAG |
| pBridge-CmPI-  CmAP3-R | T GGCTGCAGGTCGACGGATCCCTAGCCAAGCAAGGA |
| pBridge-CmPI-  CmUIF1-F | TTGACTGTATCGCCGGAATTCATGGGTGCACAGGATT |
| pBridge-CmPI-  CmUIF1-R | TGGCTGCAGGTCGACGGATCCCTATACATAAGTTTCTTTGC |

**Table S2 A list of candidate MADS-box and GARP binding motifs predicted by PlantPAN 3.0 online datebase.**

| Name | Positon | Similar score | Hit sequence |
| --- | --- | --- | --- |
| MADS-box binding motif site 1 | 101 (-) | 0.87 | TGTTTTATTTTAAGA |
| MADS-box binding motif site 2 | 270 (+) | 0.96 | TACTTAAAAAAGGG |
| MADS-box binding motif site 3 | 428 (-) | 0.74 | TTCTTTTTAGAA |
| MADS-box binding motif site 4 | 456 (+) | 0.95 | ATCTTAAATAAGTT |
| MADS-box binding motif site 5 | 1080 (+) | 0.92 | TTCTAAAATAGTA |
| MADS-box binding motif site 6 | 1214 (+) | 0.94 | TGATAAAAATAGA |
| Name | Positon | Similar score | Hit sequence |
| GARP binding motif site 1 | 334 (+) | 0.98 | TCAGATTCAG |
| GARP binding motif site 2 | 380 (+) | 0.93 | TCAGATTCAG |
| GARP binding motif site 3 | 451 (+) | 0.96 | TTGTATCTTA |
| GARP binding motif site 4 | 761 (+) | 0.97 | CAGAATCTAG |
| GARP binding motif site 5 | 780 (+) | 0.98 | CAAGATTCAT |
| GARP binding motif site 6 | 888 (+) | 0.93 | CAGGATCCCT |
| GARP binding motif site 7 | 967 (-) | 0.96 | TCAAGATATGC |
| GARP binding motif site 8 | 999 (+) | 0.96 | TGGTATCTTA |
| GARP binding motif site 9 | 1090 (+) | 0.97 | TAGTATCTTG |
| GARP binding motif site 10 | 1247 (+) | 0.90 | GGAATAATCC |

**Table S3 Yeast positive clones identified by Y1H screening.**

| **No.** | **Nr annotation** |
| --- | --- |
| 1 | MYB transcription factor HHO5-like [*Lactuca sativa*] (UIF1) |
| 2 | S-adenosyl-L-homocysteine hydrolase [*Chrysanthemum x morifolium*] |
| 3 | myb-like protein X isoform X2 [*Helianthus annuus*] |
| 4 | flower development transporter AP3 [*Artemisia annua*] (AP3) |
| 5 | acetyl-CoA acetyltransferase, cytosolic 1-like [*Helianthus annuus*] |
| 6 | ubiquitin-conjugating enzyme E2 2 [*Artemisia annua*] |
| 7 | probable carboxylesterase 18 [*Lactuca sativa*] |
| 8 | NIN-like protein [*Artemisia annua*] |
| 9 | cold-regulated 47 [*Artemisia annua*] |
| 10 | U-box domain-containing protein 15 [*Helianthus annuus*] |
| 11 | ribosomal protein S7e [*Artemisia annua*] |
| 12 | aquaporin [*Rhodobacteraceae bacterium CH30*] |
| 13 | putative alpha/Beta hydrolase fold protein [*Helianthus annuus*] |

|  | **Gene Id** | **Target gene** | **annotation** | **AP3** | **PI** | **UIF1** |
| --- | --- | --- | --- | --- | --- | --- |
| Carotenoid biosynthesis | CHR00034017 | *PSY1* | Squalene/phytoene synthase [*Cynara cardunculus*] | **-** | **+** | **-** |
|  | CHR00091590 | *PSY2* | phytoene synthase [*Chrysanthemum boreale*] | **+** | **-** | **+** |
|  | CHR00019807 | *CRTISO* | carotenoid isomerase [*Chrysanthemum* × *morifolium*] | **-** | **-** | **+** |
|  | CHR00027913 | *LCYB* | lycopene beta-cyclase [Chrysanthemum x morifolium] | **+** | **-** | **+** |
|  | CHR00027078 | *CHYE* | cytochrome P450 monooxygenase CYP97C [*Chrysanthemum* × *morifolium*] | **-** | **-** | **+** |
|  | CHR00000429 | *VDE* | violaxanthin deepoxidase [*Chrysanthemum* × *morifolium*] | **-** | **-** | **+** |
|  | CHR00026800 | *ZEP* | zeaxanthin epoxidase, chloroplastic-like isoform X4 [*Cajanus cajan*] | **+** | **-** | **+** |
| Flavonoid biosynthesis | CHR00038317 | *CHS1* | chalcone synthase, partial [*Artemisia annua*] | **+** | **-** | **-** |
|  | CHR00047213 | *CHS2* | chalcone synthase [*Carthamus tinctorius*] | **+** | **+** | **+** |
|  | CHR00008801 | *CHS3* | chalcone synthase type 4 [*Dahlia pinnata*] | **+** | **-** | **-** |
|  | CHR00036904 | *CHI* | chalcone isomerase [*Carthamus tinctorius*] | **+** | **-** | **+** |
|  | CHR00037184 | *F3H* | flavanone 3-hydroxylase [*Chrysanthemum* × *morifolium*] | **+** | **+** | **-** |
|  | CHR00000448 | *FLS* | flavonol synthase [*Carthamus tinctorius*] | **-** | **+** | **-** |
|  | CHR00050525 | *F3'H* | flavonoid 3'-hydroxylase [*Echinops bannaticus*] | **-** | **-** | **+** |
|  | CHR00058078 | *DFR* | dihydroflavonol 4-reductase [*Chrysanthemum* × *morifolium*] | **-** | **-** | **+** |
| Flower development | CHR00035692 | *CYC2a* | flower asymmetry transporter CYC2a [*Chrysanthemum* × *morifolium*] | **-** | **+** | **-** |
|  | CHR00048990 | *CYC2a* | flower asymmetry transporter CYC2a [*Chrysanthemum* × *morifolium*] | **-** | **-** | **+** |
|  | CHR00065022 | *CYC2b* | flower asymmetry transporter CYC2b [*Chrysanthemum* × *morifolium*] | **-** | **+** | **-** |
|  | CHR00093761 | *CYC2d* | flower asymmetry transporter CYC2d [*Chrysanthemum* × *morifolium*] | **-** | **-** | **+** |
|  | CHR00074698 | *CYC2f* | flower asymmetry transporter CYC2f [*Chrysanthemum* × *morifolium*] | **-** | **+** | **-** |
|  | CHR00043156 | ARF3 | Auxin response factor 3 [*Cynara cardunculus*] | **+** | **-** | **+** |
|  | CHR00001825 | ARF5 | PREDICTED: auxin response factor 5 [*Nicotiana attenuata*] | **+** | **-** | **+** |

**Table S4 Putative CmAP3/CmPI/CmUIF1-binding genes identified by DAP-Seq.**

**Table S5 Sequence entries used in this study.**

| **Name** | *CmDXS* (EVM0005576) |
| --- | --- |
| **Sequence** | ATGGCTTCATGTGGTGCTTTGAAGGGTGGATTTGCTCCAATGGCTATAGCTCAAGATGGGTTCTCTTCTTCAAATTTGCTTTGTTCATCTATCAAATCAATCGTGCCCTCGAACAAGCGAAAGTTTAAGGGAGTTGTGGCCGTTGCAAAGGACAATGCCGCTAATGACCAAGAGGACCTAAAATTAGTGAATGGGAGCGTCACAACCACCCTCAAGTACTCGGGGGAGAAACCCCAAACCCCGATATTGGATACCATTAATTATCCAGTTCACATGAAGAATCTTTGTGTCGAGGATCTCGAAAAGCTAGCTGATGAACTACGAGAGGAGATTGTATATTCAGTGTCAAAGACCGGGGGTCACTTGAGTTCGAGCTTGGGGGTCGTCGAGTTAACAGTCTCACTCCACCATGTTTTCAACACCCCAGAGACAAAATCATCTGGGATGTTGGCGTATCCACACAAAATCTTAACGGGAAGGCGGTCAAGAATGGATACAATTCGACAAACGTTTGGACTCGCAGGCTTCCCTAAGAGAGACGAGAGTCAGCATGATGCTTTTGGTGCTGGCCATAGCTCGACTAGTATTTCTGCTGGTTTAGGAATGGCTGTTGGTAGAGACTTGCTAGGAAAAGACAACCATGTAATTGCAGTGATTGGAGATGGAGCCATGACTGCAGGACAAGCATACGAGGCTATGAATAATGCTGGCTACCTTGACTCGAATCTTATTATAGTCTTGAACGACAACCGTCAAGTTTCTCTTCCCACAGCCACTCTCGATGGTCCTGCTCCACCTGTTGGCGCTCTAAGTCGATCCCTCACAAGGCTGCAAACTAGTCGCAAGTTCCGCCAACTCCGTGAAGCTGCAAAGGAAGTAACAAAGCAATTGGGTGATACAACACATGAAGTTGCATCGAAAATAGATTCTTTCGTGAAAGGAGTAGTGAGTGGTCAAGGAGCTTCCATGTTTGAAGAACTCGGGCTATACTATGTCGGTCCAGTAGATGGACACAATCTTGAGGATCTTGTCCATATTTTCAAGAAGCTTAAGTCTATGGAAACTCCTGGAGCTGTTCTGGTCCATATCGTCACAGAGAAAGGCAAAGGCTACCATCCTGCTGAAATTGCAGCCGACAAAATGCATGGTGTTGTGAAGTTCGATACTCAAACAGGAAAGCAACTGAAGGCTAAGACGAAGACACTCTCATATACTCAATATTTTGCCGATTCTTTGGTTGCTGAAGCACAGACTGATGACAAAATAGTTGCTATTCATGCGGCTATGGGAGGAGGCACAGGTCTTAACACGTTTCAAAAACACTTTCCTGAAAGGTGTTTTGATGTTGGTATTGCGGAGCAGCATGCTGTTACCTTTGCTGCAGGTTTAGCAACCGAAGGTCTTAAACCTTTTTGTGCTATATATTCATCTTTCCTCCAAAGAGGTTATGATCAGGTAGTTCATGATGTTGATCTTCAAAAGCTTCCAGTGAGATTCGCAATGGATAGGGCTGGTTTGGTTGGTGCAGATGGGCCGACTCATTGTGGTGCATTTGATACAGCTTTCATGGCTTGTTTACCAAACATGGTGGTGATGGCTCCTTCATGTGAGGCCGAGCTCATGCACATGGTTGCCACAGCTGCAGCCATTGATGATCGTCCTAGCTGCTTTAGGTACCCAAGAGGCAATGGCGTAGGCTCACCCCTCCCACCAAATAACAAAGGAAGCCCACTCGAGGTCGGCAGGGGAAGAATACTTAAGGAGGGAAGTAGAGTGGCCTTATTGGGCTATGGAACTATAGTACAAAATTGTTTAAAAGCAAACGAGCTTCTTCAAAAAATAGGTATCCAAGTGACTGTGGCAGACGCACGATTTTGCAAGCCACTGGATGGCAATTTGATCAAACAACTTGTAAAAGAGCATGAGGTCCTAATCACAATCGAAGAGGCATCTGTAGGAGGATTTAGCTCTCATGTTTCTCATTTCCTGGCCTTAAATGGATTACTTGATGGAAACCTCAAGTGGAGGGCGATGACATTACCCGATAGATACATTGACCATGGAGATCATGCTAATCAGATTGAAGAGGCCGGTCTTAGCCCAATGCATATTGCGGCAACAGTTCTATCATTGATCGGTGAGAGTAAGGAAAGACTCCTAGAAGCTGTCAATGCATAG |
| **Name** | *CmDXR* (EVM0031358) |
| **Sequence** | ATGTCTTTGAACACCCTTTCCCCTTCAGAAATCAGGGTCACTTCTTTCTTGGACACTACTAAAGCCAACACAAATTTATTTAAGCTTCAAGGTGGGGTTTCTGTAAAAAGGAAGGATAGTAAGGTAAACGGGATCCAATGTTCGGCCGCTGCATCTGGGCCGCCACCAGCATGGCCTGGGACGGCGTTGGTTCAGCCCGGGACTAAGAACTGGAGTGGGCCTAAGCCTATTTCAATTGTTGGATCCACTGGTTCTATTGGGACTCAGACATTGGACATTGTTGCTGAAAATCCTGATAAATTTAAAGTTGTAGCACTTGCTGCTGGTTCTAATGTCACACTTCTTGCGGAACAGATCAAGGCATTCAAACCACAGTTAGTCTCTATTAAAAACGAGTCGTTGGTTGCTGAACTAAAAGAAGCTTTGGCTGGTTCCGATTACATGCCTGAAATTATTCCTGGTGATGAAGGTGTTGTTGAGGTTGCTCGTCATCCAGATTGTGTTACTGTTGTCACAGGAATAGTTGGTTGTGCTGGTTTAAAGCCTACAGTTGCTGCTATTGAAGCAGGGAAAAACATTGCATTGGCAAATAAAGAAACCTTAATTGCTGGCGGTCCCTTTGTTCTTCCTCTTGCACACAAACATAATGTCAAGATTCTTCCTGCTGATTCAGAACATTCTGCTATATTTCAGTGTATACAAGGCTTCCCTGAAGGTGCTCTAAGGCGTATTATCTTGACCGCATCAGGAGGTGCTTTCAGGGACTGGCCGGTTGAAAAACTTAAAGATGTTAAAGTAGCCGATGCATTGAAACATCCAAACTGGAGCATGGGGAGAAAGATCACAGTTGACTCTGCCACACTTTTCAACAAGGGTCTAGAAGTTATTGAAGCTCATTATCTTTACGGTTCTTCGTATGACAATATTGATATAGTTATTCACCCTCAATCTATCATACATTCCATGGTCGAGACACAGGACTCGTCTGTGCTAGCACAATTAGGATGGCCCGACATGCGTTTACCAATTCTTTATACATTGTCATGGCCCGATAGAGTAGAGTGCTCGGAAATCACGTGGCCCCGTCTTGATCTTTGCAAGTTGGGTTCATTGACATTTAAAGCACCGGACAACGTGAAATACCCGTCAATGCATTTGGCGTACTCTGCTGGACGAGCTGGCGGTACAATGACCGGAGTTCTAAGTGCAGCTAATGAAAAAGCCGTTGAAATGTTTCTCGATGAAAAGATTGGCTACTTGGATATCTTTAAGGTCGTGGAGTTAACTTGTGAGAAGCATCAAGCGGAATTAGTGACAGCGCCTTCACTCGAGGAAATCATACATTATGATTTGTGGGCTCGAGAGTATGCAGCTAGTGTGAAGCCTTCATCATCTGGTTTAACTCCGGCACTAGTATGA |
| **Name** | *CmIPI* (EVM0051920) |
| **Sequence** | CTCACATGGAACATATTTTGTCCCCACTAGGTCATTTAAAAATGTAACCACCTAAAAGATTGTGACACCTTCCATAAAAATGTTAAAATTTTCTCCCTTTAAAACCATCGCCACCGCAACAAAACTTTTTCCTACACATTTCAAATCACTTCCAAAATCTCAACTCTTTCCCTCTTCAGTCCCTCTCCGCCGTCGTTACTCCGCCCTAACCGCCGCCATGGGTGATGATTCCGGCATGGATGCTGTTCAAAGACGCCTTATGTTTGATGATGAATGCATTTTGGTGGATGAGAGCGACAATGTGGTTGGGCATGATACCAAGTACAATTGCCACTTGATGGAGAAGATTGAAACAGGAAAAATGTTGCACAGAGCATTCAGTGTATTCTTGTTCAATTCAAAATACGAATTACTCCTTCAGCAACGCTCTGGAACCAAGGTGACATTTCCTTTGGTATGGACCAACACCTGTTGCAGCCATCCATTATACAGGGAATCTGAGCTTATTTCTGAAGACGTCCTTGGGGTGAGAAATGCTGCACAGAGGAAGCTCTTGGATGAACTCGGTATCCCTGCCGAAGATGTTCCAGTTGATCAGTTTACCCCTGTAAGTCGCATGCTGTACAAGGCTCCATCTGATGGGAAGTGGGGAGAACATGAACTTGACTACCTGCTCTTCATCGTGCGTGATGTTGCTGTGAACCCAAACCCAGACGAGGTGGCAGAGATCAAATATGTGAACCAAGAAGAGTTAAAAGAGTTACTAAGGAAGGCAGATGCTGGCGAGGAGGGTTTGAAACTTTCCCCTTGGTTTAGGTTAGTCGTTGACAACTTCTTGTACAAATGGTGGGATCATGTTCAAAAGGGTTCTCTCTCTGAAGCCATTGATATGAAAACCATCCATAAGTTGCTTTAAGAAAAACCCACTCAAATGACATGGTGTTATGCTTTGAAGACCTCACCACCTCTGTTTCATTATTCCTTTTAGAAAGCTTTATCTACATCTCACCATGTTCTGTGCAACGTATTACTTTTGGTCTTTCGTTTGAAGAGTGCAATAACGAACCGCTTGATAGTTCGGAACTTTGAAATTTACAGTTGCTTTTTACCTTTAATTTAAATGTATTTTAGCCCTCAATGATGATGTGAATGAAGTATATCATAATTTGGGATTGAATTGCTCCTTTGATGTGTTTCAATCTAAAAGAGTATAATGCATATGACTTGAGCTTTGCAAACAACTGGATTGTGGTTTGGGACTTTTGTAAATGATCATTAGAGGCCGTTTAGTCCACCGATGTCAGATGGATCACTATAATTACAAGC |
| **Name** | *CmGGPS* (EVM0014663) |
| **Sequence** | TTACTCCCTCTTTAGCAAATTTATCAACTATGGTTCCTGCAGCTCATATTTCTATCTCGAGAATCAATGTAAGCCATCGATCGAGCTATCTTGCCAGGACTATCGGGTAAGCGTCAATACACGACCGTTGCTCGTGTGGCCCAAAATCAATCATATTGGGAGTCGATACAATCTGATATCGACTCCCACTTGAAAAACGCAATACCAATCCGCAAGCCTTTAACTGTCTTTGAGCCAATGCACCATTTAACATTCTCACCACCAAGAACAAGTGCTTCTGCATTATGTGTTGCCGCCTGTGAGTTAGTAGGCGGCAACAGGACCGACGCTATTGTGGCAGCGTCAGCTATTCATCTCATGCATGCAGATATGTACGTGCATGAGCATATGCTGTTAACTGACAGGGTTGAACCCCAACCCACAATCCCACACAAGTTTGACCCGAAAATCGAGCTTATGACTGGTGATGGTATTATGCCATTTGGGTTTGAGTTACTAGCTGGGTCTATGGACCCAGCTTGTAACAATGCTGGCAAAATACTAAGAGTAATAATTGAGATCTCAAGAGCTATAGGCTCGGAGGCAATGATTAGTGATAAAGATTACGAAGCTGATATTGACCAATCGAGTGGTCAACAAATTCATCGGCTCCACGAAAGTGGGGCCGCGTGTGGAGCAATACTAGGAGGTGGGAATGAAGAGGAGATCGAGAGGCTAAAAAAGTTCGGACTATATGCAGGCAAAATACAAGGACTACTAAATAAAATGGGTAAAAATGAGGACGGTAAAATCAAGTTAATTGAGAAATGGAGAGCTTTGGCTTTGAAGGAGTTGGAACATTTTGACGGTAAAAAAATTGAGCAAATTTCTACAATTGTGACCGGTGATAGTCTAAAATTTTCTTATGTTGGTAAGTCATCATTTGTTGTGTCATGTTAA |
| **Name** | *CmPSY1* (EVM0027963) |
| **Sequence** | ATGTCAGGTGCTATGATTTGGGTTATTTCTCCTAATTCTGATATATGTAATGGTTTGGA  AAAAAGGGTTTTAATGAGTGCAAAATTTAAGAATGTTGGTAAGAAAATTTTAAGGAGATTAGAAGTGAGTACAAAAGTAGTGGCTAATACAACAGGGAACTTGGCTATTTCTTCTGAGCAATTGGTTTATGATGTTGTGTTAAAGCAAGCTGCTTTGGTTAAGAAGCAGCTAAGAAATAGGGAGGAAGAAGATATGGATGTTAAGCCTGAAATTGTGCTGCCTGGTACACTTGGGTTGTTGAATGAGGCTTATGATAGATGTGGTGAAGTTTGTGCTGAGTATGCTAAAACTTTTTATTTAGGAACGTTGTTGATGACACCCGAGAGGCGAAAAGCTATATGGGCGATCTATGTATGGTGCAGGAGAACAGATGAACTCGTTGATGGACCTAATGCATCACATATAACACCCAAAGCTCTAGATCGATGGGAGTCGAGATTAGAAGATCTTTTCAATGGGCGTCCTTTTGATATGCTCGATGCTGCTTTATCAGATACTGTTTCTAAGTTTCCTGTTGACATTCAGCCATTTAAGGATATGATCGATGGAATGAGGATGGACCTTAAAAAGTCAAGATACGAGAACTTTGATGAGCTTTATCTTTATTGCTATTATGTAGCGGGAACAGTTGGCTTGATGAGTGTTCCAATAATGGGAATCGCTCGTGAATCGAATGCACCAACAGAAAGTGTTTACAATGCTGCTTTGGCTTTAGGGATTGCTAATCAACTCACAAACATCCTTAGAGATGTAGGAGAAGGAAGAGGAAGAGTTTACCTGCCACAAGATGGACTAGCGCAAGCTGGATTATAGAATGAAGATATATTTGCAATGAATGTAACTGATAAATGGAGACTATTCATGAGAAAACAAATAAAGCGTGCAAGAACATTCTTTGACCAAGCAGAAGAAGGAGTTACACAACTAGATTCTGTTAAGAAAATGAAAATGATCAAGACACTCATTCGTCTTTA |
| **Name** | *CmPSY2* (EVM0033788) |
| **Sequence** | ATGTCTGTTGCTTTGATATGGGTTGTTTCACCTACTTCTGAGCTTAGTAATGGGCTGGGATTCTTGGATACAACAAAGTTAGTTGATGCATCAAAACCGAGAAGCGTTTTTAAGTCTAGCAGAATCAAGAATGTTGGTAAGAAGTACAAGCACAAGTGCAAGTCATGTTATGTTAATGCAGATTTGAAGTTTGGTGCTTCGGGTTTAAATAATGGGAGAAAATCGCAGTTTGATGTTTCGAGGGTGGTGGCTAATCCAACTGGTGAATTGGCTATTTCGTCAGAGCAATTGGTTTATGATGTGGTTTTAAAGCAAGCTGCGTTGGTTAAGAAACAGATGAAAGGTAAAGAAGAAGAAGAAGATATGGAAGTGAAACCGGATATTGTGCTTCCTGGAACGCTTGGTTTATTGAGTGAAGCTTATGATAGATGTGGTGAAGTTTGTGCAGAATATGCAAAGACATTTTATTTAGGAACATTGCTAATGACACCGGAAAGGCGAAAAGCTATATGGGCGATTTACGTATGGTGTAGGAGAACCGATGAACTTGTTGACGGGCCTAATGCATCACATATAACACCAAAAGCTCTAGATAGATGGGAGTCAAGGCTAGAAGATCTTTTCAATGGTCGCCCTTTTGATATGCTTGATGCTGCTTTATCAGACACTGTCTCCAAGTTCCCAGTTGACATTCAGCCTTTTAAGGATATGATTGAAGGAATGAGGATGGACCTTAGAAAGTCAAGATACAAGACTTTTGATGAGCTTTATTTGTATTGCTATTATGTGGCTGGTACAGTCGGATTGATGAGTGTTCCTATAATGGGAATTGCTCCCGATTCCCAAGCATCAACAGAAAGTGTATACAATGCTGCTTTGGCTTTAGGAATCGCTAATCAACTCACTAACATACTTCGAGATGTGGGAGAAGATGCAAGAAGAGGAAGAGTGTATCTACCACAAGATGAACTAGCACAAGCCGGACTATCAGATGAGGATATATTTGCTGGAAAAGTAACCGATAAATGGAGGATTTTTATGAAAAGGCAAATTAAACGGGCAAGAGCCTTCTTTGATGAAGCACAAGAAGGCGTCACTCAACTAAGCTCAGCTAGCAGATGGCCAGTGTGGGCATCCCTATTACTGTACCGTCAAATCTTGGATGAGATTGAAGCCAATGACTATAACAACTTCACTAGAAGAGCTTATGTAAGTAAACCGAAGAAGCTAGTATCTTTACCACTTGCTTATGCTAAGTCTCTTGTTCCTCCATCAAGAAGTGGAATTCTTTCGAAGACCATGGACGTGTAG |
| **Name** | *CmPDS* (EVM0005023) |
| **Sequence** | ATGATGTCTCTGTCCGGAAATGTCTCCGCCTCTTTAGTTTCAACCATCACTTCCCCCCGAGATTCTATTAATACTTCTTCTGCCAAACTACTTTCATTTGGACGAGCTGATTCTATCATTGGCCACCAGTTCCCACTAGCTCGTTCATATTCAACCAAGAGGGGGAGGAATGTAACCCCTTTAAAGGTTGTTTGCGTTGACTATCCTCGACCTGACCTTGACAACACCTCTAATTTCTTGGAAGCTGCTTACTTGTCTTCTACCTTCCGTGCCTCCCCACGTCCATCTAAGCCGTTGAACATCGTCATTGCTGGTGCAGGTCTTGCCGGTTTATCAACTGCTAAGTATTTGGCTGATGCCGGTCACAAGCCTATTTTGCTAGAAGCAAGAGACGTTCTCGGTGGAAAGGTGGCGGCTTGGAAAGACGAGGATGGAGATTGGTACGAGACGGGTTTACACATATTTTTTGGAGCTTACCCAAACGTGCAGAACCTGTTTGGAGAGTTAGGCATTAATGATAGATTGCAGTGGAAGGAACATTCTATGATATTTGCGATTGCAAACAAGCCTGGAGAATTTAGTCGCTTTGACTTCCCAGATGTTCTGCCTGCACCATTGAATGGAATATGGGCTATCTTGAGGAACAATGAAATGCTGACATGGCCGGAGAAAGTAAAATTTGCAATTGGGCTCTTGCCTGCAATGTTAGGTGGACAGGCGTATGTTGAGGCTCAAGATGGGCTAAGTGTTCAAGACTGGATGAGACAGCGAGGCATACCGGATCGAGTTACCACCGAGGTGTTTATTGCCATGTCAAAGGCATTAAACTTCATAAATCCTGATGAACTGTCAATGCAATGTATTCTCATAGCTCTTAACCGGTTTCTTCAGGAAAAGCATGGTTCTAAGATGGCGTTTTTAGATGGCAGCCCTCCTGAGAGACTTTGCAGGCCAATTGTCGAGCATATTGAGTCGTTAGGTGGCCAAGTCAGGCTTAATTCACGAATTCAAAAGATTGAGTTAAACAAAGATGGAACTGTCAGGAACTTTCTACTGAATGATGGGAATATTATCAAAGGTGATGCCTATGTATTCGCTGCTCCAGTTGACATTCTGAAGCTTCTTTTGCCGGAGGATTGGAAACCAATTCCGTACTTCAAGAAGCTGGATAAATTAGTTGGTGTTCCAGTTATAAACGTTCACATATGGTTTGACAGGAAACTCCGAAACACATACGATCACCTACTCTTCAGCAGGAGTCCTCTTCTCAGTGTATATGCTGACATGTCTGTAACATGTAAGGAATATTATGATCCAAACCGGTCCATGTTGGAGTTGGTTTTTGCACCAGCAGAAGAATGGATTTCACGAAGTGACTCTGATATTATTGATGCAACGATGAGTGAACTTTCAAGACTCTTTCCTGATGAAATTGCAGCAGATCAGAGCAAAGCGAAAATATTGAAATACCATGTAGTTAAAACACCAAGGTCTGTTTACAAAACAGTACCAGACTGTGAACCTTGCCGTCCATTGCAAAGATCTCCAATAGAAGGATTCTATTTAGCTGGAGACTACACGAAACAAAAGTATTTGGCTTCAATGGAAGGTGCCGTTCTATCTGGAAAATTTTGCGCCCAAGCTATTGTACAGGATTATGACTTGCTTGCGGCCAGGGGGGAAGTGGTTGCTGAGGCGAGTCTTGTCTAA |
| **Name** | *CmZDS* (EVM0015447) |
| **Sequence** | ATGACAAAACACTCACCACCAGCATCTCCCCATTTCCTGATTCTGTTATCTCACATCCCTCCTCTCCTCACCTAATTTATACCTAAATTTTATTAATCCCATTACATTACATATCATTTCACTCACACACACACACTAAAACAAGTGTTAGGTGTTAAAAGTGTGTGTGTGTATGTGAGTTAGTTATATGATGGCTACATCTTCCACGTCATCACTCTGTTTCCCGGCAACTTCTGCTGCCGGTACACGTCACTCTTTTCCGACTAACGATACTTTCTTACGGTGTAACCGTCATCGTAACCGTCGGTTGAAGTTTGTTGTGAGGTCTGATTTGGATAAAGATGTTTCCGATATGAGAACTAATGCTCCAAAAGGATTATTTCCACCCGAGCCTGAACATTATCGTGGGCCCAAGTTGAAAGTGGCCATCATAGGTGCTGGCCTTGCTGGTATGTCAACTGCGGTGGAGCTTTTGGATCAAGGACATGAGGTGGATATATACGAGTCAAGGACGTTCATTGGTGGAAAAGTGGGTTCATTTGTTGATAAAGGTGGAAACCATATTGAAATGGGACTACATGTTTTCTTTGGTTGCTACAACAATCTTTTTCGTCTTTTGAAGAAGGTTGGTGCTGAGAAGAACCTTCTTGTCAAGGATCATACTCACACTTTTGTGAACAGGGGAGGAGAAATTGGCGAACTTGATTTCCGCTTTCCTGTTGGAGCACCATTACATGGGATTAACGCATTCTTAACTACAAATCAGCTTAAGACATATGATAAAGCGAGAAATGCTGTGGCTCTTGCTCTAAGTCCAGTTGTGAGGGCGCTTGTTGACCCAGATGGAGCAATGAACCAAATACGGAACCTTGATAATGTTAGCTTCTCTGAATGGTTTATGTCCAGAGGAGGAACACGAGCGAGTATTCAGAGAATGTGGGACCCTGTTGCTTATGCTCTTGGATTTATTGATTGTGATAACATCAGTGCTCGCTGTATGCTCACCATTTTCTCATTATTTGCCACCAAGACAGAGGCCTCTTTGTTGCGCATGCTTAAGGGTTCTCCAGATGTCTATCTCAGTGGTCCTATCAGAGATTATATTATCGAGAAAGGAGGAAGGTTCCATCTCAGATGGGGGTGCCGGGAGATTCTCTACGAAAAATCAGATAATGGAGATACTTATGTTACAGGGCTTGCGATGTCTAAGGCTACTCAAAAGAAAATTGTGAAGGCAGATGCATATATAGCAGCATGTGACGTCCCTGGAATCAAAAGACTTTTACCTTCTAGTTGGAGGGATTGGGAGTTCTTTGATGATATCTACAAACTAGTCGGTGTTCCTGTTGTGACTGTGCAACTTCGGTACAATGGCTGGGTAACAGAAATGCAGGATATAGAACGAGCCAGGCAATCAAGGAAAGCTACAGGGTTGGACAATCTACTATATACCCCAGACGCAGACTTTTCTTGTTTTGCTGACTTGGCACTGACCTCACCAGAAGATTACTATATTGATGGACAAGGCTCATTGCTGCAATGTGTGCTGACTCCAGGGGACCCATACATGCCTTTACCAAATGAAGAAATTATAAGACGGGTTACAGAACAGGTTTTGGTTCTATTCCCATCTTCCCAAGGTCTAGAAGTTACTTGGTCATCTGTGGTCAAGATTGCTCAATCTTTATATCGAGAGGGACCTGGTAAAGATCCATTTAGACCTGATCAAAAGACACCAGTCAAGAATTTCTTCTTGGCTGGTTCATACACAAAACAGGACTACATAGACAGCATGGAAGGGGCAACTTTATCAGGCAGACAAGCTTCTGCTTTTATATGTGATGCCGGGGAAGAACTTGCAGCCTTGAGGAAACAACTTGCTGCTGTTGAATCCGTAGGTACCATTGGAGTTGATGAGCTTACTCTTGTTTGAGGCATTGCTAGTTACAGGATTATAGTGTATGTTTCTTGTATAATTGATTGGGAAAATGTAAGAAAATACACTATCAATATATCATTTTAATTATAAAAAGAAACTACAGTTTA |
| **Name** | *CmLCYB* (EVM0028157) |
| **Sequence** | TTGAAGATAAACAAAATGCCGTCATGAGACATCCACAAAATTACATTTCTTTATCCATCTCCAAAAAACCATGATTTCTCTTCACCCCTCAAAAGTAAGTCTTTCCCTTTTTCTTCCTTTCAATTCACCCAATTTTAGATAATATTTTATTCAATTAGTACTTAAAGTTTGTAACTTTATTGTGTTTTTTAGTTAAAATTGCATCTGGGTATGATGGATACTTTGTTAAAGATTCATAATTCATTCGAATTTGTGCACCCAATTAAAAGATTTGCAGGAAATGTGAATGCTTTAAGCTCCAAAAAGCCTTATTTCAAATGGGGTTATAATAATTATAATAAAGATTGTGTTAAAGCTAGTGGCAGTAGTGCTTTATTAGAGCTTGTACCCGAAATCAAGAAAGAAAACCTTGATTTTGAGCTTCCTATGTATGATTATACAAAAGGTGTTGTGGTGGATTTAGTTGTGGTTGGTGGTGGTCCTTCGGGTTTAGCTGTAGCTCAGCAAGTGTCCGAGGCTGGGCTTACGGTTTGCTCGATTGACCCATCTCCGAAATTGATTTGGCCGAATAATTATGGTGTTTGGGTGGATGAATTTGAGGCTATGGACTTGTTGGATTGTTTAGATACTACTTGGGCTAGCGCGGTTGTTTATATTGATGAAAACTCGACTAAGAGTCTTAATAGGCCTTATGCGAGGGTTAATAGGAAACATCTTAAGACTAAGATGTTACAGAAGTGTATATCAAATGGGGTTAAGTTTCATCAGGCGAAAGTGATTAAAGTTGTTCATGAGGAATTTAAGTCGTTGTTGATTTGCAATGATGGTGTGACAATTCAAGCGACTTTGGTTCTTGATGCAACGGGGTTTTCAAGATCTCTTGTTCAGTATGATAAGCCTTATAATCCGGGGTATCAAGTGGCGTATGGTATTTTAGCAGAAGTAGAGGAACACCCTTTTGATGTCGATAAAATGCTCTTCATGGATTGGAGAGATTCACATCTTGGAAAAAATACCGAAATAAAGGAAAGGAATTCAAAGATTCCTACTTTTCTTTACGCGATGCCCTTTTCGTCTAATAGGATATTTCTTGAAGAAACTTCACTTGTGGCTCGACCGGGGTTAAAAATGGAAGATATACAAGAAAGAATGGTGTGCCGGTTAAAGCATTTGGGTATAAAAGTGAAAAGTATTGAAGAAGATGAGCGGTGTGTTATCCCAATGGGCGGGCCCCTTCCTGTTTTGCCTCAAAGAGTTCTTGGAATTGGTGGGACCGCCGGGATGGTTCATCCTTCGACCGGATATATGGTGGCAAGAACTTTAGCTGCTGCCCCAATTGTTGCAAAATCAATAATTCAGTATCTTAATTCAGAAAAAATGCTGTGTGGAACAGATTTATCAGCAGAAGTTTGGAGGGATTTGTGGCCTATAGAGCGGAGAAGACAACGAGAATTCTTCTGTTTTGGTATGGATATCTTGCTAAAGCTCGATTTAGAAGGCACAAGACGGTTTTTTGATGCATTTTTTGATTTAGAACCTCGTTACTGGCATGGGTTCTTGTCATCAAGATTGTTTCTACCTGAGTTATTGACTTTTGGTCTTTCTCTTTTTGCTCATGCTTCTAATACTTCTAGAATAGAGATTATGGCAAAAGGAACTCTCCCGTTGGCAAACATGATCAACAACTTAGTTCAAGATCGAGATTAAATATTAAGATTCATACATTATACTTGTAAGCTTTCATTTTTCACGATCTGGATATTATGATTATTGTATGTTTGAGTTGA |
| **Name** | *CmLCYE* (EVM0039265) |
| **Sequence** | ATGCAACAGAATAAGTCTATGGATTCTCAGTCCAGCTTGTCCGAAAAGTTGCCACGGATACCGATTGGAGAGTGTATATTGGATTTGGTTGTAATCGGTTGTGGTCCTGCTGGTCTTGCTCTTGCTGCTGAATCAGCTAAGCTTGGTCTGAATGTCGGACTTATTGGACCTGATCTTCCTTTTACGAATAATTACGGTGTTTGGGAGGATGAATTTATAGGTCTTGGACTTGAAGGGTGTATTGAACATGTTTGGCGGGATACTATCGTATATCTTGATGAAAACGATCCCATCCTCATAGGCCGTGCCTATGGACGAGTTAGTCGTGATTTACTTCATGAGGAGTTGTTAAGAAGGTGTGTGGAGTCAGGTGTTTCATATCTGAGTTCCAAAGTGGAAAGAATTACTGAAGCTACAAACGGCAACAGTCTCATCGAGTGTGAAGGCAATATTATCGTTCCGTGCAGGCTTGCTACTGTTGCATCTGGAGCGGCTTCGGGTAAACTTCTGCAATATGAGCTTGGGGGTCCTCGTGTTTGTGTTCAAACAGCTTACGGTGTAGAGGTCGAGGTTGAAAGCATTCCCTATGATCCAAGCCTTATGGTTTTCATGGATTATCGAGATTATGCAAAACATTCGGCTGAATCTGTAGAAGCACAATATCCGACATTCCTTTATGTCATGCCTATGTCTCCAACCAAAGTATTTTTTGAGGAAACCTGTCTGGCTTCAAGAGAGGCTATGCCGTTTAATCTACTGAAGACAAAACTCATGTCGAGATTAAAGACAATGGGGGTCAAAATAACCAAAGTTTATGAAGAGGAATGGTCGTATATTCCAGTAGGCGGATCATTACCAAATACAGAACAAAAGAACCTTGCGTTTGGTGCTGCTGCTAGCATGGTGCATCCAGCCACAGGCTATTCAGTAGTCAGATCGCTGTCAGAAGCTCCCAACTATGCAGCAGCAATTGCAAAGATATTACAGCAAGGACGTTCCAAACAGATGCTTGATCTCGGAAGATATACAACTAACATTTCAAAGCAAGCGTGGGAAACCCTTTGGCCATTGGAAAGGAAAAGACAACGAGCATTCTTTCTCTTTGGCTTAGCACTGATCGTGCAACTGGATATTGA |
| **Name** | *CmCHYB* (EVM0048287) |
| **Sequence** | ATGGCGGCAGCTATTGCTGTCCCTTGCAGCTCACGCTCATTCAAACTAACCCGAATGCCATTGGGTCAAAAACCCATGTCACACTTTCCATCTTTAGTCAGAAAATTTGACCCACTTGTGAGATATCGTAGGATGCCTCCTAGCTTGACTGTGTGTTTTGTGGCTGGAGATAAACAAGTTGAGACCACCAAATCTGTGGCTGGTGATGGTCCAGGGATTAGTAGTCGAGGGTGAAGGGGATGTGATAAGGATTAGTACTAGTGTTGAGGAGAAAATGAAGAGAAAAAAGTCTGAAAAGTTTACTTATCTTGTTGCTGCTATTATGTCTACTTTTGGGATTACTTCTATGGCTGTTATGGCTGTTTATTATCGCTTTTCTTGGCAAATGGAGGGTGGAGAGATTCCTTATGTAGAGATGTTCGGCACATTTGCACTCTCTGTTGGTGCTGCGGTAGGAATGGAGT  ATTGGGCTAGATGGGCGCATGAAGCGCTATGGCATGCTTCATTGTGGCACATGCATGAGTCGCATCATAAACCACGAGAGGGTCCATTTGAGCTCAACGACGTGTTTGCAATCATAAACGCCGTGCCAGCAATTGCTTTACTCAACTATGGTTTCTTCCACAAAGGCATAATTCCCGGTCTATGTTTTGGTGCGGGTTTGGGGATAACTGTGTTTGGTATGGCTTACATGTTCGTCCACGATGGACTTGTTCATAAGAGATCCCAAGTGGGTCCCATTGCAAATGTTCCCTACCTTCGAAAAGTTGCAGCTGCTCATCAGCTGCATCACACGGAGAAATTCAACGGAGTTCCTTATGGCTTGTTCTTGGGACCTAAGGAGCTCGAAGAAGTAGGAGGAATGGATGAGTTGGAGAAAGAGATCCAAAGAAGGATCAAGTTGTCTAAAAAATAA |
| **Name** | *CmCHYE* (EVM0040022) |
| **Sequence** | ATGCCAACCATACTACACTCACTCTCACCCCTCATAACAACAACCACCACACACCACCATATCACCACCAAAAACCACCACCCAAATACCTACCCCACTTCACCATCAAATTCAAAAAACGGGTCATGGGTCAGCCCAGACTGGCTAACATCACTAACAAGGTCACTAACCTTCACAAAACAAGACAACTCAAACATACCAATAGCAGAAGCAAAACTAGAAGATGTATCTGATCTTTTAGGTGGGGCCCTTTTTTTACCTTTGTTTAAATGGATGAATGATTATGGACCTATTTATAGGTATGACCCATTAATTGCTAAACATGTTTTGAGAAACTATGGTACTATATATGCTAAAGGTCTTGTTGCTGAGGTTTCTGAGTTCTTGTTTGGTTCCGGTTTTGCTATTGCTGAAGGTTCTCTTTGGACGGCAAGGCGCAGGGCTGTGGTTCCATCGCTTCACAAGAAATACCTATCAGTAATAGTTGATCGTGTATTTTGCAAATGCTCCGAAAGATTTGTGGACAAGCTCAAAACGTATGCACTCAATGGCACTGCTGTCAACATGGAGGAACAATTTTCTCAGTTAACGCTTGATGTTATTGGTTTGGCGGTATTTAACTACAATTTTGATTCACTCACGTCTGATAGTCCTGTGATTGAATCTGTTTACACCGCACTAAAAGAAGCTGAAGCTCGTTCAACTGATCTGTTACCTTATTGGAAGATTGATGCATTATGCAAAATCATCCCAAGACAAATAAAAGCCCAACAAGCAGTTACTATAATTAGAGAAACTGTTGAAGAACTTATATTGAAATGCAAGGAGATGGTTGAAAAAGAAGGCGAAAAAATTGATGATGAAGATTATGTGAATGATGCCGATCCAAGCATCCTTCGATTCTTGCTTGCTAGCAGAGAAGAGGTAACGAGTCAACAGCTTCGTGATGACCTTTTGTCAATGTTGGTTGCTGGACATGAAACCACTGGTTCCGTGTTAACTTGGACTGCATATCTTTTAAGTAAGGATCCATCTTCTTTAGTGAAGGCTCAAGAAGAAGTTGACAGAGTGTTACAAGGCCGACCGCCAACATACGAAGACATAAAGAGCCTCAAGTTTATAACCCGATGCATAAATGAGTCAATGCGTCTCTACCCACATCCTCCGGTATTAATAAGAAGAGCTATAGTCGCGGATGAGCTTCCAGGAAATTATAAGGTCAATCCGGGTCAAGATATTATGATATCGGTGTATAACATCCATCATTCTTCCCAGGTATGGGAAAGAGCAGAAGAATTCATACCTGAAAGATTTGACTTGGATGGGCCTGTGCCAAACGAAGCCAACACCGATTACAAGTACATACCGTTCAGTGGTGGCCCACGGAAATGTGTAGGTGATCAGTTCGCTATGATGGAAGCGATTGTTGCTCTTGCAATATTTTTACAGAAAATGGAGTTCGAGTTGGTTCCTGATCAAAAAATTAACATGACAACGGGCGCCACCATTCACACAACAAGCGGTTTGTACATGAAAGTCAAAGAACGCCAAGTCAATTCTATGATGGTCTCTTCTGTATAG |
| **Name** | *CmVDE* (EVM0007951) |
| **Sequence** | ATGGCTCTTTCTTTGCACACTGTATTTCTCTGCAAAGAGGAGACTGCTAGTTTGTATGCCAGATTACCAACTCATGAAAGGTCTCACAGGATTGGACCACCACCTCCTGCCAGTATAGTTACGATGAAAATTCGATCTACCCACGGGTTTTATAAACTATTTCAGTGGTTTAGATCTTGTAGGATTAGTTACTTACCGGACTCTAAGTATTCAACTCCATTGTTACATTGCAAGATGAAATCTCCAGTATGTAGCATTGATGAAAGCTTTGAGGAAATAGAAAATTTTGATCTTAGAAAGGGTGTACCTTTGATTTTCAAAAAGCAATGGAGGCAATTCATACAATTGGCTATCGTATTGGCTTGCACATTTGTTGTCATTCCCAGTGTTGATGCTGTTGATGCTCTTAAAACATGTACTTGCTTACTGAAAGAATGCAGGATCGAGCTTGCGAAATGTATAGCGAACCCATCTTGTGCAGCAAATGTTGCCTGTCTCCAGACTTGCAACAATAGACCAGATGAGACCGAATGTCAGATAAAATGTGGCGACTTGTTTGAAAACAGTGTTGTGGATCAGTTCAATGAGTGTGCAGTATCACGAAAGAAATGTGTGCCACGCAAATCAGATGTTGGTGAATTTCCAGTCCCGGATCCTAGTGCGCTTGTCAAAAGCTTCAATATGAATGATTTCACTGGGAAGTGGTACATAACAAGTGGTCTAAATCCTACATTTGATGTCTTTGATTGTCAACTGCATGAATTCCACACGGAAGCTAATAAGCTAGTAGGGAATTTAACGTGGCGAATAAAAACTCCAGATGGCGGATTCTTTACTCGATCTGCTGTTCAAACGTTTGTGCAAGATCCGAACCAACCTGGAGTTCTATACAATCATGATAATGAGTTTCTTCACTATCAAGATGACTGGTACATCTTGTCTTCCCAAGTTAACAACAAACCAGACGACTACATATTTGTATATTACCGTGGAAGAAACGATGCATGGGATGGATACGGTGGAGCCGTAATTTACACAAGAAGTTCAAAACTACCACAAACCATCATCCCACAGCTACAACAAGCAGCTAAAAGCGTTGGACGTGACTTCAACACTTTCATCGAAACAGATAACACTTGCGGGCCCGAACCACCACTAGTGGAGAGACTAGAAAAGAAAGCCGAAGAAGGTGAAAAGTTCATCATCAAAGAAGCCGAAGAGATTGAAGAGGAGGTCGAGAAAGAGGTGGCCAAGGTCAGAGACGCGGAAATGACATTGTTTCAGAGGTTAGTCGAAGGATTTAAAGAGTTGCAACAAGATGAACAAAGCTTCCTTAGGGAGCTCACTAAAGAAGAGAAGGAAATCTTGGATGAACTTTCAATGGAAGCTACTGAAGTCGAAAAACTTTTTGGGCGTGCTTTACCAATTAGAAAATTAAGGTAA |
| **Name** | *CmZEP* (EVM0021607) |
| **Sequence** | ATGGCAACTTCACATGTATATTGCAGCCCAACAATTACACTTCACTCTAAAACAAACTTGCCAGTTGAATTCTTTAGCTCAATACATTATAAACACCATCATCTCAAAAGTAAAGAAAATGGGTCATTTAAGAAACTTACTAATTATGGAAAGATCAAAGCCTTAGTGACTGAAACCCCACCGCCAAAAACGGAGCAAAGTGGTGGTGAGAAAGAGAAGAAGATAAGGGTATTGGTGGCAGGTGGTGGAATTGGTGGATTAGTGTTTGCATTAGCTGCTAAAAGAAAAGGATTTGAAGTTGTGGTGTTTGAAAAAGATTTGAGTGCTATTAGAGGTGAAGGTCAATATAGAGGACCTATTCAGATACAAAGTAATGCTTTGGCTGCTTTGGAAGCTATTGATTTAGATGTTGCTGATGAAGTTATGAAAGCTGGTTGTATTACTGGTCAGAGGATTAATGGCTTGGTTGATGGCATTTCTGGTAATTGGTATATCAAGTTTGATACATTCACTCCAGCAGTCGAAAGAGGTCTTCCGGTTACTAGAGTTATAAGCCGAATGACTTTACAAAAAATTCTAGCTGATGCTGTAGGCGACGAAATTATTTTGAATGGTAGCAATGTAGTCGATTTTGAAGATCATGGTGACAAGGTTTCCGTGGTTCTTGAAAACGGAGAACGTTTTGAAGGAGATCTTCTAGTTGGTGCTGATGGTATATGGTCTAAGGTACGGAAGAATTTGTTTGGGCCAAAGGATGTTACTTACTCTGGCTACACTTGTTACACTGGGATTGCTGATTTTATACCACCTGATATTAACTCTGTTGGATATCGGGTGTTCTTAGGCCATAAACAATATTTTGTATCTTCTGATGTTGGTGGTGGAAAGATGCAATGGTATGCATTTCACAATGAACCAGCTGGTGGTTCTGATAAGCCAAATGGTAAAAAGGAACGACTTCTTGAAATCTTTGGAGGTTGGTGTGATAACGTAGTGGATCTGTTGCTTGCCACTGATGAAGAAGCAATTTTAAGGCGGGATATATTTGACCGGATTCCAAAGTTTACATGGGGAAAGGGCCGTATAACTTTGCTTGGAGACTCGGTCCACGCAATGCAGCCTAACTTGGGTCAAGGAGGTTGCATGGCTATTGAGGATAGCTATCAACTAGCACTTGAGCTAGATAAAGCTTGGATACAAAGCACCAAGTCGGGAGCACCTATAGATATCCAATCTTCCCTTAGGAGATATGAAAATGCCAGAAGACTACGGGTTGCAGTCATACATGGATTGGCAAGAATGGCAGCAATCATGGCATCAACTTACAAGGCTTATTTGGGTGTGGGGCTTGGTCCATTGTCGTTCTTGACTAATTTCAGAATACCACATCCTGGACGAGTTGGTGGAAGATTTTTCATTGATATTGGAATGCCTTTGATGCTCAGTTGGGTTCTTGGTGGCAATGGCTCAAAACTTGAAGGGAGACCACAGTCCTGCAGACTCACCGATAAAGCAAATGATGAACTTCAAAACTGGTTTAGAGATGATGATGCTTTAGAACGTGCTCTTACTGGCGAATGGTTTTTACTTCCAATTGGAAGTTCAAATGCTGATTCAGCGCCTGTCTCATTAAGCCGTGACGAGAAAATGCCCTGCATAGTTGGGAGCGTGCCGCACACGAGTATCCCTGGAAACTCAATAGTCATCTCTTCTCCAGAGATTTCAAAACTGCATGCTCGAATAAGCTGCAAAGATGGTGCCTTTTATGTTACTGATTTGCGCAGTGAACATGGTACCTGGATCACAGATAACGAGGATAGGCGATATCGGGTGCCTCCAAATTTCCCAGCACGTTTTCATCCATCAGATGTCCTAGAGTTTGGACCAAATAAAAAGGTAGCGTTTCGTGTAAAGGTGATTAGATCCCAACCAAAGATTACCGAGGAGGGAGGAGACAGAGTTCTCCAGGCAGTATGA |
| **Name** | *CmCCD1* (EVM0003018) |
| **Sequence** | ATGGGGTCAATGGCGACGGAGATAAGTGGTAACGGAGTAGTGACAGTTAATCCAAAGCCTAGCAAAGGCTTCACTTCGAAAGCAGTGGATTGGATTGAGAAGGTAGTAGTGAAGCTAATGTATGATTCATCACAGCCTCATCATTGGTTGTTTGGTAACTTTGCACCTGTTGATGAAACTCCTCCTTGTAAAGACCTCGATGTTATCGGTCACTTACCGGAGTGCTTGAATGGAGAGTTTGTTAGAGTAGGTCCCAATCCGAAGTTTGCTCCTGTGGCTGGATATCACTGGTTTGATGGAGATGGTATGATCCATGGCTTGCGCATCAAAGATGGAAAAGCAACTTATCTCTCACGTTATGTGAAGACATCCCGTTTGGAACAGGAAGAATATTTTGGAGGAGCAAAGTTTATGAAGATTGGAGATCTTAAAGGGTTTTTTGGATTAGTGATGGTCCAAATGCAAATGCTTAGAACAAAGTTGAAAGTGCTAGATCTTACATATGGCCAGGGGACAGGTAACACAGCTCTCGTATATCATCATGGAAAGCTATTGGCACTATCAGAAGGAGATAAACCATACGCCATAAAAGTTCTGGAGGATGGTGACCTGCAAACTCTTGGCTTGATGGATTATGATAAGAGGCTTTCTCACACATTCACCGCTCACCCTAAGATTGATCCAGTCACTGGTGAAATGTTTGCGTTTGGATATGCACAAACACCACCATATTGCACATACCGTGTGATTTCAAAGGATGGTGTGATGAGTGATCCGGTGCCAATAACAATACCAGAATCAGTGATGATGCACGACTTTGCTATTACTGAGAATTATGCAATCTTCATGGATCTACCTTTGTACTTTAGACCAAAAGACATGGTGAAGGAGAAAAAGTTTGTTTTCACATTTGATGCAACAAAGAAAGCTCGATTTGGTATGCTTCCTCGGTATGCAAAGAATGAGCTCCAGATTAAATGGTTTGAGCTCCCAAATTGCTTTATATTTCACAACGCCAATGCATGGGAAGAAGGAGAGGAAGTTGTTTTGATTACATGTCGCGTTCAAAATCCAAATTTGGATAAGGTCGGTGGAGGTGTTGAGGAAAAGCGTGAAAATTTTTCAAATGAGCTGTATGAGATGAGATTCAACATGGAAACTGGTCTTGCTACTCAGAAAAAGTTATCAGAGTCTGCTGTTGATTTTCCAAGGGTGAACGAGTACTACACTGGCAGGAAACAACGATTTGTGTATGCAACTAGGCTTGACAGCATTGCAAAGGTTACTGGGTTTGTAAAGTTTGATTTACATGCAGAACCCGAGGCTGGAAAAGAAAAAATAGAAATTGGAGGAAATGTTCAAGGCATGTATGATCTGGGCCCCGGTAGATATGGTTCAGAGGCAATATTTGTTCCTAAGCAACCAGGAGTTGACTCTGAAGAAGACGATGGCTACTTGATCTTCTTTACACATGATGAGAACACAGAGAAATCATCAGTGACAGTTATAGATGCGAAAACAATGTCTCCAGATCCTGTTGCAGTTGTTCCATTGCCCCATAGAGTTCCATATGGTTTTCATGCTTTCTTTGTGACAGAGGAGCAAATTCAGGAACAAGCAAAATTCTGA |
| **Name** | *CmCCD4a-2* (EVM0019666) |
| **Sequence** | ATGGGCTCTTTTCCCATATCTCTTCTGTCAACATTTTTAAGACCAAATTCCATCCCATTTCAACATCAACCACGACGACGACCACCAGCACCACCACCGTCATTACCCCAGCCATCAGCTTGTCTTGTCTTTTCTGCCAGAATAGAAGAAAATCAACCAACCGTCACAACCACCAGGAGGCCTAAAAGAAAATGGCCGAAGAAGCTAATATCTTCAACTCGTAAACACCGAACAAAATCAGTGAAGGAAGATCAGCCCTTACCAACCATGATCTTCAAAGTTTTTGATGACATCATAAATAACTTCATTGATCCACCACTTAGGGTTTCGGTTGACCCAAAACATGTCTTATCACATAACTTTTCTCCTGTTAACGAACTCCCTCCAACTGAATGTGAAATCATTGAGGGCATACTTCCTAGTTGCCTTGATGGAGCATACTTCCGTAACGGGCCAAATCCACAGTATCTACCACGGGGTCCCTACCACATCTTTGACGGTGATGGCATGCTTCACGCTATTCGTATCTCAAAAGGAAGAGCCACATTTTGTAGTCGATATGTGAAAACTTACAAGTACCAGACAGAGAAAGATGCGGAATCACCCATCTTTCCAAATTTGTTTTCAGGGTTCAATGGTATGACTGCCTCTATTGCTCGATTGGCAGTTAGTACAGGACGGATTTTGATGGGCCAGTTTGACCCAACAAAAGGTATTGGTGTTGCAAATACTAGCATCGCATATTTTGGCAATAAACTTTATGCATTAGGGGAGTCAGATCTCCCCTATGCCATCAAACTTGCAGCCAATGGTGATATAATCACCATTGGACGTGATGACTTTGACGGTAAGCTGTTAACCAATATGACTGCTCACCCAAAACTAGATCCGGTAACTAAAGAAACTTTTGCTTTCCGGTACGGCCCCGGGCCTCCTTTCTTAACATTTTTCTGGTTCAATGAAAATGGAAAAAAACAGGATGATGTTCCAATCTTTTCAGTGATAAGCCCCTCTTTTATTCACGACTTTGCCATCACTAAAAACTATGCAATTTTCCCTGAAAATCAAATTGAAATGAGTCTAATGGGGATGATTGGAGGTGGATCACCAGTTAGGGCAGACCCCAGGAAGGTTGCGCGAATTGGGGTGATTACAAGGTATGCCAAGGATGATTCAGAGATGAAGTGGTTCGAGGTTCCAGGGCTTAACGTTGTACATTGCATTAGCGCATGGGAAGAGGATAACGGAGATACTATTGTGATGGTGGCGCCTAATATATTATCTGTAGAGCACGCTTTGGAGAGGATGGATTTGGTCCATGCATCCGTAGAGAAAGTGACAATGGATCTGAAAAGTGGGATGGTTTCACGGTACCCTCTTTCAACCCGGAATCTTGATTTTGCAGTCATCAACCCGGCTTTCGTCTCTGTAAAAAACAGGTATATTTATTGTGGAGTCGGGGATCCAATGCCTAAAATCTCGGGGGTCGTAAAGCTAGACATTTCATTATCTGAAGTTGACCATCGCGAGTGTATTGTGGCTAGCCGAATGTTTGGGCATGGTTGTTTTGGCGGTGAGCCATTCTTTGTGGCTAAGGAACCCGAAAATCCATATGCAGGCGAGGATGATGGTTACATAGTTTCTTATGTGCATAATGAGATCACTGATGTGTCCAGATTTGTGGTGATGGATGCCAAGTCGCCTACACTAGAGATAGTAGCTGCTGTGAAACTGCCCCACCGGGTGCCCTATGGCTTCCACGGTCTCTTCGTTAGGGAAAAATACCTAACAACGCTTTGA |
| **Name** | *CmAP3* |
| **Sequence** | ATGGCTAGAGGAAAGATCCAGATCAAGAAGATCGAGAACTCGACGAATAGGCAGGTCACGTATTCGAAGAGGAGGAACGGGTTGTTCAAGAAGGCGAGTGAGCTTACGGTGTTGTGCGATGCTAAGGTTTCGATTATTATGGTGTCGTGTACTGATAAGCTTCATGAGTACATAAGTCCTTCCATTACGACAAAGCAGTTCTTCGATCAGTATCAAAAGGCATCTGGAATTGATCTATGGAACTCTCATTATGAGAAAATGCAAGAGGTACTGAGGCAGCTGAAAGATGTGAACAAAAAACTTAGAACGCATATCAGGCAAAGGTTGGGTGATTGTTTGGATCATCTTGGTTTTGAAGAACTTCTTGATCTTGAAAAGGAGTCTCAGGAGGCTGTCTATGTCATTCGTGAACGCAAGCTCAAAGTGATCGGTAATAAGCTGGAGACTTCCAAGAAGAAGGTCAGAAGTGCTCAAGACGTATACAAGAAGCTAATGCATGAATTTGATATAAGGGGGGAGGATCCGCAATTTGGGATGATAGAAGATGCAGGAGAGTACGAAACCATCTATGGATACCCACCACATATCGCTACACAACGCATACTCACTCTACGCCTGCAGCCTAACCATCCAGATAATCTCCATGCTGCTACTGCATCAGACCTCACCACCTATTCCTTGCTTGGCTAG |
| **Name** | *CmPI* |
| **Sequence** | ATGGGCACGGGAAAGATAGAGATCAAGAGGATAGAAAACACAAGTAACAGACAAGTCACATATTCAAAAAGAAAGAATGGAATCATCAAGAAAGCTAAAGAAATAACTGTTCTTTGTGATGCTAATGTCTCTCTTGTTATCTATGGATCTTCTGGCAAGATGTATGAGTACTGCAGCCCTAAAACTAATTTGATTGACATGCTGGATCGATATCAAAGACTTTCTGGAAACAAGTTGTGGGATGCTAAACATGAGAATCTTCAGAATGAAATTGATAGAATCAAGAAAGAGAATGAAAGCATGCAAATAGAGCTCAGGCACTTGAAGGGAGAAGATATCACATCTTTGAACTATGAAGAACTAATTGCGTATGAAGATGCACTTGAAAATGGACTCACCAATATCCGTGAGAAAAAGGATGACATCCCCAAAATCATGAGGAAGCATGAGCAAGTTTTGGAGGAGGAAAATAAGCATCTTATGTATTTAGTGCAACAAAGTGAAATGGCAGCCATGGGAGATTACCAAGCTCATGAACCCTTTTCATTTCGTGTCCAGCCAATGCAGCCTAACTTGCATGAGAGGATGTGA |
| **Name** | *CmUIF1* |
| **Sequence** | ATGGGTGCACAGGATTTGAGTTTAGATTTGAATCCAGTTTTTATACCAAAAACGATCAGTCAGTTTCTAGGTGATTTAATGAATATGAATTCTGTGACTGAAAAGATATTAGCAGTTGATGATTATGTTCATAGATTGGAAACTGAAATTATTAAAATCCATGCTTTTGAACGTGAACTGCCCTTGTGTAAGCTCTTGATGAATGATGCAATTGTGGCTTTGAAAGAGGAGTTAATGGTGTTTAGAAGATCAGTAAATGCCCCAGTGATTGAAGAGTTTATACCAATTAAGAAAAGCTGTGCTGATGATGATGAGGTGTCGAAATTCGAAAGCGATATAGGTGATAAGAAGAATTGGCTGAGTTCTACTCAGCTTTGCACGAATCCGAAACAAAGTTTCGAGCAAGTGGTTAAAAAGCGAAGCGAAGAACGAGATGTGATGGTTAAAAAGTTTATCCCGTTCAAGGAGGACATGCCGGTTCCTGGCCTTTCGCTTATTAGTCCTGGAATTAAGAATCCAATGAGGGGAAATGCTTATCTTGCGAAGAATGGTGCTAGTGATAAACTGGTTTCTTATACTATCCGGGATGTTCAATCGGATATACAGATTGGTGGTATGAAGTCTCAGCCAACACTACAACCGACGTCTAGGAAACAAAGAAGATGCTGGTCTCCGGAGTTGCATAGACGCTTTGTTAATGCATTGCAACAACTTGGTGGTTCAAAAGTAGCCACACCTAAGCAAATTCGAGAGCTTATGCAAGTAGATGGTCTCACAAATGACGAAGTAAAGAGCCATCTGCAAAAATACCGTCTTCATAACAAAAGATTTCCATCTGGTGTTACTTCGGGAGCTTCATGGGCTCAGTCACATGATCATTATCTTGAAAAACATTCAAATTCTCAGTCGGGTTCTCCTGATGGACCGCTCCTGAACTACACCACCGGTGGTACTTCAACCACTGGTGGCGACAGTATGGATGACTGTGAAGATGAGAGATCCGAGAACAATTGCTGGAAAGGTCATCTTTACACCTCAGGCAAAGAAACTTATGTATAG |
